# Role of Ki-67 and Annexin V in the Biological Behavior of Salivary Gland Tumors: Insights into Proliferation and Apoptosis

**DOI:** 10.3390/cimb48040387

**Published:** 2026-04-10

**Authors:** Balkees Taha Garib, Dalya Ali Abdulla

**Affiliations:** Department of Oral and Maxillofacial Pathology, College of Dentistry, University of Sulaimani, Madam Mitterrand Street, Sulaimaniyah 46001, Iraq; balkees.garib@univsul.edu.iq

**Keywords:** Ki-67, annexin V, salivary gland tumors, proliferation, apoptosis

## Abstract

Salivary gland tumors have diverse biological behaviors, and the exact molecular factors underlying their pathogenesis remain unclear. The expression of Annexin V and its potential association with Ki-67 in these tumors has not been explored. Therefore, this study aimed to evaluate the immunohistochemical expression of Ki-67 and Annexin V and to assess their relationship in salivary gland tumors. This study included 45 formalin-fixed, paraffin-embedded blocks (5 normal salivary gland tissues, 10 pleomorphic adenomas, 10 Warthin tumors, 10 mucoepidermoid carcinomas, and 10 adenoid cystic carcinomas). Immunohistochemical staining for Ki-67 and Annexin V was performed and evaluated semi-quantitatively. Depending on the results of the normality test, one-way ANOVA or the Kruskal-Wallis H test was used for group comparisons. Spearman’s rho test was used to assess correlations among the markers under study. A *p*-value < 0.05 was considered statistically significant. Both markers and their ratio showed statistically significant differences among the groups (*p*-value < 0.001). Normal salivary gland tissue and pleomorphic adenoma showed negative Ki-67 expression, whereas Warthin tumor, mucoepidermoid carcinoma, and adenoid cystic carcinoma showed weak proliferation indices. Annexin V expression was highest in the normal salivary gland tissue. Within individual tumor types, Ki-67 and Annexin V exhibited no significant correlation. The combined evaluation of Ki-67 and Annexin V expression, along with their relationship, may provide preliminary insights into the biological behavior of salivary gland tumors and warrant further clinicopathological investigation.

## 1. Introduction

Salivary gland tumors (SGTs) constitute a rare and diverse group of benign and malignant neoplasms characterized by complex, heterogeneous histology [1]. Despite advances in molecular techniques for understanding the pathogenesis of various tumor types, several aspects of SGTs remain underexplored [2]. An important component in tumor biology is the assessment of cellular proliferation and death, as these processes significantly influence the tumor growth dynamics [3].

Ki-67 is widely recognized as the most commonly used marker for evaluating cellular proliferation because it is expressed in all active phases of the cell cycle [4]. It is considered a simple, rapid, and reliable means of assessing tumor proliferation [4]. Ki-67 has been studied in SGTs and been shown to have negative or low expression in benign tumors, indicating slow cellular proliferation and generally indolent behavior [5]. In contrast, malignant tumors display variable Ki-67 proliferation indices (PIs) ranging from low to high expression depending on tumor type and histologic grade. Elevated Ki-67 expression is generally associated with higher grades, recurrence, and a poor prognosis, making it a valuable marker of tumor aggressiveness [5,6]. However, a detailed analysis of its proliferative pattern, including its regional distribution and cellular type/components, has not yet been thoroughly investigated.

On the other hand, a variety of markers are available for the study of apoptosis, among which Annexin V (ANX-V) is widely used due to its selective binding to phosphatidyl serine and frequent application across tumor types [7]. ANX-V is a calcium-dependent protein expressed intracellularly and extracellularly, where it plays key roles in membrane organization (endocytosis, exocytosis, and repair), apoptosis, and inflammation [8,9]. It can localize to different cellular compartments, including the nucleus, cytoplasm, and membrane, in response to various cellular functions [10,11]. This property allows detection by immunohistochemistry (IHC), enabling visualization of the marker’s distribution across tissue structures, cell layers, and tumor components. Although ANX-V is classically known as an apoptosis probe in flow cytometry, previous studies have demonstrated that ANX-V can also be detected in formalin-fixed, paraffin-embedded (FFPE) human tissues by IHC [12,13]. Immunohistochemical expression of ANX-V has been reported in a variety of tumors, including invasive ductal carcinoma and fibroadenoma of the breast, colon cancer, gastric cancer, cervical cancer, and ovarian cancer, where its expression was correlated with clinical parameters such as tumor stage, progression, and metastasis [12,13,14,15].

On the other hand, several members of the Annexin family have been investigated in SGTs, including Annexin I (ANX-I), which has been identified in proteomic analyses of pleomorphic adenoma (PA) and Warthin tumor (WT), and is thought to be associated with pathways related to apoptosis and tumor progression [16]. Likewise, Annexin II (ANX-II) is significantly overexpressed in malignant SGTs and is suggested to correlate with invasive behavior [17]. However, based on the existing literature, the role of ANX-V in the SGTs remains uninvestigated.

This study aims to evaluate IHC staining for Ki-67 and ANX-V in four types of SGTs to characterize their proliferative and apoptotic profiles. We hypothesized that SGTs exhibit variation in Ki-67 and ANX-V expression across tumor types, reflecting differences in their biological behavior.

## 2. Materials and Methods

### 2.1. Study Design and Ethical Approval

This retrospective cross-sectional study was conducted at the University of Sulaimani/College of Dentistry, Department of Oral Pathology, from November 2024 to July 2025. It was approved by the local Ethics Committee (code: COD-EC-24-0053; Ethics Committee, College of Dentistry, University of Sulaimani) on 16 December 2023. The study protocol was in accordance with the Declaration of Helsinki.

### 2.2. Sample Size Calculation, Case Collection, and Data Collection

The sample size was calculated using G*Power software (Version 3.1; Heinrich Heine University Düsseldorf, Düsseldorf, Germany). Based on data from a previous study [6], an effect size (Cohen’s f = 0.80) was estimated and applied to an F-test model with α = 0.05, power = 0.80, and four groups. The required minimum sample size was 34 cases (~9 per group).

Therefore, a total of 45 FFPE salivary gland tissue blocks were retrieved from the pathology archives of five hospitals in Sulaimani city between 2014 and 2024 using a predetermined balanced design. They included 20 benign tumors (10 PA and 10 WT), 20 malignant tumors (10 mucoepidermoid carcinomas [MEC] and 10 adenoid cystic carcinomas [AdCC]), and 5 morphologically normal salivary gland (NSG) tissues as a control. The clinicopathological data (age, sex, site, tumor size, recurrent status, and survival outcome) were obtained from their reports (survival data were collected from patient follow-up records at the time of data collection in December 2024). Cases were excluded if they had insufficient tissue, poor fixation, or missing substantial clinical data, withdrawn samples, or the absence of clearly identifiable peripheral tumor regions. Information regarding prior chemotherapy or radiotherapy was not consistently available; therefore, pretreated cases were not specifically excluded.

The diagnoses of the cases were confirmed independently by two pathologists using hematoxylin and eosin-stained slides. Discrepancies were resolved by consensus.

### 2.3. Immunohistochemistry (IHC) Procedure

Serial 4 μm-thickness tissue sections were cut and mounted on positively charged slides. The slides were deparaffinized in xylene and rehydrated through a graded ethanol series (100%, 90%, 70%) to distilled water. Antigen retrieval was performed using Dako Target Retrieval Solution (pH 6.0 and pH 9.0) in a PT Link (Dako, Agilent Technologies, Santa Clara, CA, USA) system at 97 °C for 40 min, followed by cooling to room temperature.

After 2 rinses of the slides with phosphate-buffered saline for 2–3 min (the same rinsing process was performed at each washing step throughout the staining procedure), tissue borders were defined with a Pap pen. Endogenous peroxidase activity was blocked using peroxidase-blocking reagent (Dako, SM801) for 5–10 min. Non-specific binding sites were then blocked with serum-free protein blocker for 10 min. Sections were incubated in humidified incubation chambers (45 min at room temperature) with the following primary antibodies:

• Mouse monoclonal IgG, anti-Ki67 antibody, dilution 1:100 (ab16667);
• Rabbit polyclonal IgG, anti-Annexin V antibody, dilution 1:1500 (ab140068).

Slides were washed and incubated with a secondary antibody (Dako EnVision Flex HRP, SM802) for 45 min at room temperature. Color development was performed using DAB+ chromogen in substrate buffer (Dako, SM803), yielding a brown reaction product at antigen-positive sites. Sections were counterstained with Mayer’s hematoxylin for 2 min, dehydrated, cleared in xylene, and covered with DPX mounting medium and cover slips.

Positive and negative control tissues were included in each staining batch to ensure specificity and staining quality. Human tonsil tissue was used as a positive control for Ki-67, and human breast adenocarcinoma was used as a positive control for ANX-V. Negative control was obtained by omission of the primary antibody.

Representative positive and negative control images are provided in Supplementary File S1 (Figure S1–S4).

### 2.4. Interpretation of Staining and Scoring

The stained slides were evaluated under a conventional light microscope. Microphotographs were captured using a smartphone (iPhone 11 Pro Max, Apple Inc., Cupertino, CA, USA) mounted on a microscope adapter to ensure stability and image clarity.

For each case, 10 high-power, ‘hot-spot’ fields were selected within the representative tumor areas. Initial screening was performed at ×40 magnification to identify areas with the highest marker expression, followed by detailed evaluation at ×400 magnification. Only cells with nuclear staining were considered Ki-67-positive. Any cell exhibiting membranous, cytoplasmic, or nuclear staining was counted as total ANX-V positive. In addition, nuclear ANX-V was defined as distinct brown staining in the nucleus and perinuclear region. Individual tumor cells were manually selected and digitally marked using an image analysis tool (CountThings by Camera by Precision, Version: 1.6.6). The software visually labels each selected cell to prevent duplication and automatically generates the total cell count based on the marked cells. Cell counting was performed by a single observer who was blinded to the clinical and pathological data at two separate time points. The mean of the two readings was used for analysis. Ten randomly selected fields from all groups were evaluated, and reliability was subsequently assessed statistically.

In each case, more than 1000 tumor cells were evaluated across the selected fields. The PI and ANX-V expressions were quantified by calculating the percentage of positively stained cells relative to the total number of counted tumor cells. The final value for each case was obtained by averaging the results across the examined fields. This counting method followed the approach described by Bussari et al. [6] using the following formula:

$$\mathrm{Percentage}\;\mathrm{of}\;\mathrm{positive}\;\mathrm{cells}\;=\frac{\left(Number\;of\;positive\;cells\right)}{\left(Total\;number\;of\;cells\right)}\;*100$$

This counting approach was consistently applied to relevant tissue components in PA and to the epithelial layers in WT for Ki-67 and ANX-V nuclear localization. For benign tumors, spatial expression patterns were documented as peripheral or central distribution. For malignant tumors, histological grades for MEC and histological patterns for AdCC were considered. In AdCC, marker expression was assessed separately for each pattern by averaging across selected fields. The mean expression was grouped in 4 scales: negative 0–5%, weak 6–25%, moderate 26–50%, and strong 51–100% [18].

### 2.5. Statistical Analysis

Data were entered into a Microsoft Excel spreadsheet and analyzed using SPSS (IBM SPSS Statistics, V27, IBM Corp., Armonk, NY, USA). Descriptive statistics were used to summarize data. Intra-observer reliability was assessed by the intraclass correlation coefficient (ICC), which demonstrated excellent agreement (ICC = 0.941; 95% CI: 0.781–0.985), with a statistically significant F-test (F = 37.845, *p* < 0.001).

Continuous variables are presented as the mean ± standard deviation (SD), and categorical variables are expressed as frequencies and percentages. Normality test was performed using the Shapiro-–Wilk test, while homogeneity of variances was evaluated using Levene’s test. Based on the normality test, the comparison of continuous variables among groups was performed using either a one-way ANOVA or the Kruskal–Wallis H test. When statistically significant differences were detected, Dunn’s post hoc test was applied for multiple pairwise comparisons between groups. Comparisons of categorial variables were conducted using Fisher’s exact test, as more than 20% of cells had expected frequencies less than 5. The correlation between the expression level of markers was assessed using Spearman’s (rho) correlation test. The point-biserial correlation coefficient (rpb) was used to assess the relationship between markers and binary clinical parameters, including recurrence and survival outcome. For subgroup analyses involving very small sample sizes (*n* < 5), statistical comparisons were not performed due to the limited reliability of statistical tests. In such cases, the data were presented descriptively (mean ± SD) to illustrate expression trends. A *p*-value < 0.05 was considered statistically significant.

## 3. Results

### 3.1. Demographic Features

In this study, 40 tumors were included, with patients aged 21–80 years (mean ± SD: 48 ± 14 years). There was no significant difference among tumor types (one-way ANOVA, *p* = 0.086). Overall, and across all malignant tumor types, this study observed a male predominance (*n* = 25, 62.5%); this sex distribution was statistically significant by Fisher’s exact test (*p* = 0.046). Tumor site distribution also differed significantly among tumor types (*p* < 0.001), with the parotid gland being the most commonly affected site for benign SGTs. Approximately half of the PA cases occurred in the parotid gland, whereas all cases of WT were exclusively located in the parotid gland. Malignant neoplasms were frequently found to be presented in the minor SGs (MEC—70%, and AdCC—80%). The mean size of the total sample was 3.25 ± 2 cm and was relatively similar across tumor types. Recurrence was observed in 7 cases (17.5%) and did not differ significantly between tumor types (*p* = 0.109). At the most recent evaluation, outcome data were available for 30 cases, with follow-up duration varying between cases. Among these, 28 patients (93%) were alive, and 2 patients (7%) had died (both deaths occurred in AdCC cases). Survival outcome differed significantly between tumor types (Fisher’s Exact test, *p* = 0.036). Detailed demographic characteristics are summarized in Table 1.

### 3.2. Ki-67 and Annexin V Immunohistochemistry

**Figure 1:**
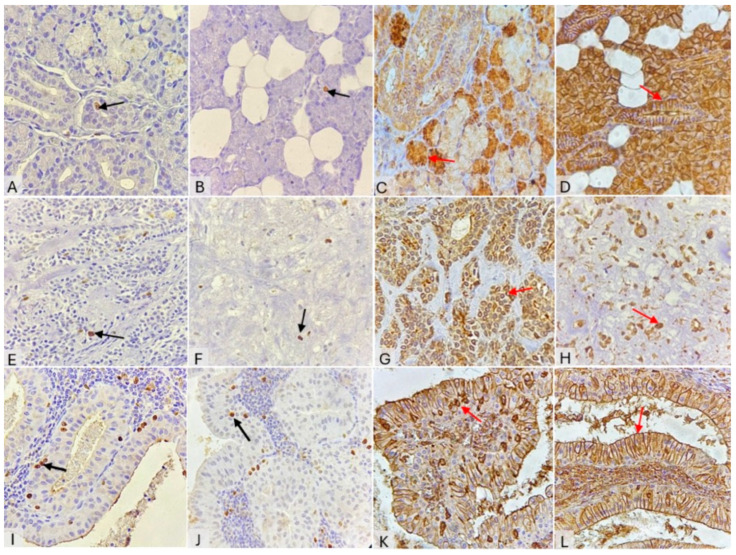
IHC expression of Ki-67 (black arrows) and ANX-V (red arrows) in normal salivary gland tissue and benign salivary gland tumors, magnification ×400. NSG: Ki-67 expression (**A**,**B**); ANX-V expression (**C**,**D**). PA: Ki-67 immunoreactivity in the epithelial component (**E**); Ki-67 in the mesenchymal components (**F**). ANX-V in the epithelial component (**G**); ANX-V in the mesenchymal component (**H**). WT: Ki-67 expression in basal oncocytes (**I**,**J**). ANX-V expression in basal oncocytes (**K**); ANX-V in the luminal layer (**L**).

All 45 cases of SG samples exhibited positive expression for both Ki-67 and ANX-V. Ki-67 showed nuclear expression, while ANX-V was detected in the nuclear/perinuclear region, cytoplasm, interproximal membrane, or any combination of these sites. NSGs revealed sparse Ki-67–positive cells, limited to ductal and acinar cells (Figure 1A,B). In contrast to Ki-67, ANX-V showed strong positivity in NSGs, observed in both ductal and serous cells (mucous cells were negative). In striated ducts (SD), ANX-V demonstrated a distinct distribution pattern, being localized along the intercellular membranes and concentrated in the basal striation near the membrane–stromal junction (Figure 1C,D).

In benign tumors, PA showed low Ki-67 immunoreactivity. The majority of dividing cells were located within the epithelial component (Figure 1E,F). In contrast, overall ANX-V expression was prominent; most of the ANX-V-positive cells showed nuclear (perinuclear) localization and were predominantly observed in the mesenchymal component. Cytoplasmic and membranous ANX-V expression was mainly detected within the epithelial component (Figure 1G,H).

Ki-67 immunoreactivity within the bi-layered epithelial lining of WT appeared overall low, and basal oncocytes more frequently exhibited Ki-67 staining than luminal oncocytes (Figure 1I,J), whereas ANX-V expression was highly distinctive and uniformly distributed across the epithelial component of WT. One frequently observed pattern involved luminal oncocytes, in which staining sharply outlined the apical and interproximal membranes of nearly all columnar cells. Meanwhile, nuclear ANX-V expression was confined to the basal cells, with occasional cytoplasmic staining observed in some oncocytes. This expression pattern of ANX-V in WT closely resembled its expression in the SDs of NSGs. However, in the basal cells, the preferential cytoplasmic staining of ANX-V in the membrane–stroma interface was diminished or absent, with immunoreactivity appearing either focally concentrated or showing apparent nuclear translocation (Figure 1K,L).

**Figure 2:**
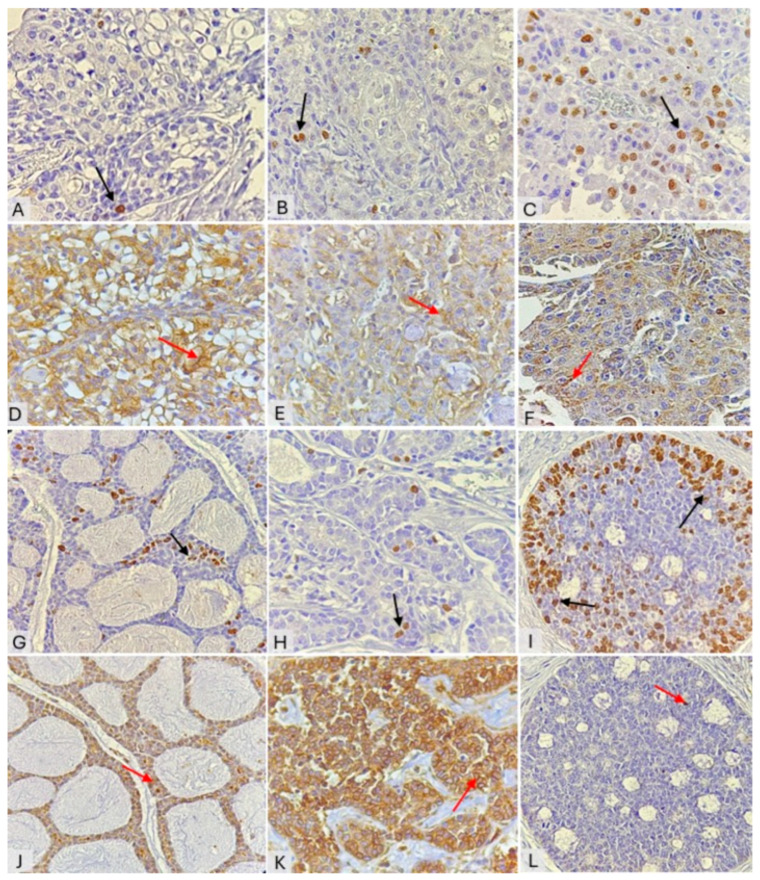
IHC expression of Ki-67 (black arrows) and ANX-V (red arrows) in malignant SGTs, magnification ×400. Ki-67 expression in MEC: low-grade (**A**); intermediate-grade (**B**); high-grade (**C**). ANX-V expression in MEC: low-grade (**D**); intermediate-grade (**E**); high-grade MEC (**F**). Ki-67 expression in AdCC patterns: cribriform (**G**); tubular (**H**); solid (**I**), ANX-V expression in AdCC patterns: cribriform (**J**); tubular (**K**); solid (**L**).

Malignant SGTs exhibited variable expression of Ki-67 and ANX-V. Depending on the histological morphology, proliferative activity was most prominent in high-grade MECs and was primarily restricted to epidermoid cells across all grades of MEC (Figure 2A–C). In contrast, ANX-V appeared mainly as cytoplasmic, membrane, and occasionally nuclear staining in MECs. The highest ANX-V expression was observed in low-grade tumors, mainly in the membranes of mucous and clear cells, and in the cytoplasm of epidermoid cells in high-grade tumors. Intermediate-grade MECs showed slight membrane staining (Figure 2D–F).

On the other hand, AdCC showed overall Ki-67 expression. In all three histologic patterns, most of the proliferation was observed in the solid pattern, and the peripheral cells of solid islands harbored more Ki-67 positivity than the central cell areas (Figure 2G–I). ANX-V generally showed low AdCC activity. The highest ANX-V immunoreactivity was observed in a tubular pattern, appearing as membranous and perinuclear expression. The cribriform pattern typically revealed partial membranous and cytoplasmic staining, while the solid pattern exhibited slight to negative ANX-V expression (Figure 2J–L).

Representative photomicrographs are presented in the main figures, while complete microscopic fields of the same cases and additional sections are provided in Supplementary File S2 (Figures S5–S32).

### 3.3. Quantification (Scoring) and Statistics

**Figure 3:**
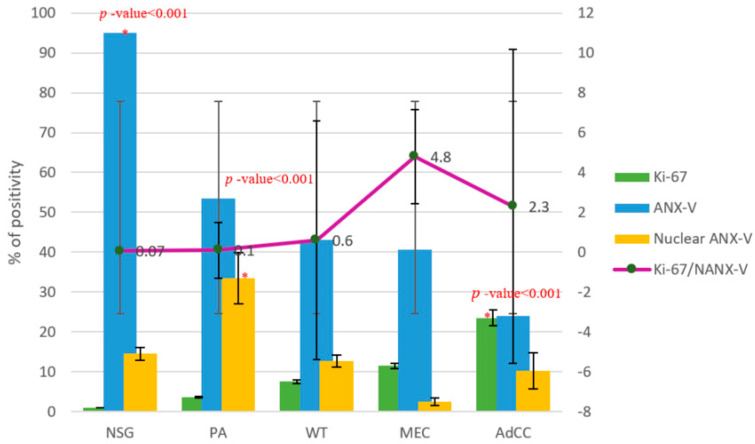
A representative bar chart shows the expression levels of Ki-67, ANX-V, and nuclear ANX-V in NSGs and benign and malignant SGTs. (*) indicates a statistically significant difference.

NSGs revealed only 1 ± 0.18% Ki-67 positivity and were scored as negative. In contrast to Ki-67, total ANX-V showed strong positivity (95 ± 1.39%) in NSGs and weak nuclear ANX-V (14.5 ± 1.67%) expression (Figure 3). A statistically significant difference in ANX-V expression was observed among the five groups (Kruskal–Wallis H = 20.908, *p*-value < 0.001). With a large effect size (ε^2^ = 0.42). Dunn’s post hoc test revealed that NSGs differed significantly from all other groups (*p*-value < 0.001).

In the benign tumors, PA showed a Ki-67 labeling index of 3.3 ± 0.48% and was therefore scored as negative (Figure 3). Comparable PI levels were observed in both the peripheral (3.2%) and central zones (3.4%) of the lesion. Of the total Ki-67 positivity in PA, 86% was localized within the epithelial component (Table 2). On the other hand, total ANX-V expression was strong, accounting for 53.5 ± 6% of the tumor (Figure 3), with uniform distribution across the peripheral and central regions. Additionally, 80% of ANX-V-positive cells were located in the mesenchymal component (Table 2). PA exhibited the highest nuclear ANX-V expression among all groups, with a mean value of 33.5 ± 6.4% (moderate score). The Kruskal–Wallis H test revealed a statistically significant difference in nuclear ANX-V expression among the five groups (H = 23.846, *p*-value < 0.001), with a large effect size (ε^2^ = 0.47) (Figure 3). Dunn’s post hoc test revealed significant pairwise differences between groups (*p*-value < 0.05).

Ki-67 immunoreactivity within the WT was weakly expressed (7.5 ± 0.68%) (Figure 3), approximately 86% of which was located in the basal layer, and evenly distributed between the peripheral and central regions (Table 2). It had moderate total ANX-V expression (43 ± 2.36%), with slightly higher positivity in the central region (45%) than in the peripheral region. Of the total ANX-V, 66% was expressed in the luminal oncocytes. Meanwhile, weak nuclear ANX-V localization was observed, accounting for 12.7% (Figure 3).

Regarding malignant SGTs, AdCC showed the highest Ki-67 expression (23 ± 4.4%, H = 33.625, *p*-value < 0.001), with a large effect size (ε^2^ = 0.74), followed by MEC (11.7 ± 2%) (Figure 3); both were scored as weak. Dunn’s post hoc test revealed that AdCC differed significantly from all other groups (*p*-value < 0.05). Among the MEC grades, high-grade tumors exhibited the highest Ki-67 expression, accounting for 18% (Table 2). In the histological patterns of MEC, the solid pattern showed the highest Ki-67 expression (37%), falling within the moderate score range (Table 2). Total ANX-V was moderately expressed in MEC (40.6 ± 7.89%), and low-grade tumors showed approximately 55% ANX-V reactivity. However, nuclear ANX-V was nearly absent in MEC, accounting for only 2.4 ± 0.95% (Figure 3). Meanwhile, AdCC exhibited weak total ANX-V and nuclear ANX-V expressions, accounting for 24 ± 1.3% and 10.25 ± 4.57%, respectively (Figure 3). Among the histologic patterns, the tubular-type exhibited the highest ANX-V immunoreactivity (33%) (Table 2).

The proportion of cellular proliferation relative to apoptosis (Ki-67: nuclear ANX ratio) in the salivary gland (SG) samples revealed that NSG demonstrated the lowest ratio (0.07), followed by PA (0.1), and WT (0.6) (Figure 3). In contrast, this ratio increased markedly in malignant tumors, reaching a peak in MEC (4.8) and (2.3) in AdCC (Figure 3). Spearman’s (rho) correlation analysis revealed a significant inverse relationship between Ki-67 and nuclear ANX-V expression when all tumors were combined (Spearman’s ρ = −0.538, *p* < 0.001). However, stratified analyses showed no statistically significant correlation within any individual tumor type: PA (Spearman’s ρ = 0.335, *p* = 0.343), WT (Spearman’s ρ = 0.086, *p* = 0.813), MEC (Spearman’s ρ = −0.226, *p* = 0.531), or AdCC (Spearman’s ρ = 0.140, *p* = 0.700).

The relationship between Ki-67 and ANX-V expression with recurrent status and survival outcome is summarized in Table 3. Correlation analysis revealed no statistically significant relationship between either Ki-67 or ANX-V expression and recurrence status (*p* = 0.251 and *p* = 0.222, respectively). In contrast, both markers demonstrated significant correlations with survival outcome. Ki-67 showed a moderate positive correlation with survival outcome (r = 0.402, *p* = 0.028). Conversely, ANX-V exhibited a moderate negative correlation with survival outcome (r = −0.421, *p* = 0.021), suggesting reduced ANX-V activity in cases associated with mortality.

## 4. Discussion

Salivary gland neoplasms are rare and heterogeneous tumors; in our community, they account of 18.7% from the total registered oral and maxillofacial surgical biopsies [19]. Despite studies on the biological differences among SGTs, these differences remain under further investigation.

Alongside IHC analysis, summarizing the clinicopathological and demographic features helps contextualize molecular findings. The mean age of patients was 48 ± 14 years, similar to that reported by Mohammad et al. [20] and consistent with other Iraqi and international studies reporting peak incidence in the 40–50 year age group [21,22]. In this study, PA was more common in females, whereas WT predominated in males, with the parotid gland being the most frequently affected site. Malignant SGTs showed a male predilection and were more commonly observed in the minor SGs. These findings are consistent with previous reports in the literature [21,22]. The mean tumor size in the present study was 3.25 cm across all four SGT types, with no significant difference between them. This aligns with the results of Ungureanu et al., indicating that most SG neoplasms present at a size of 2–4 cm at diagnosis [23]. Recurrence was observed in PA (10%), MEC (20%), and AdCC (40%), which is relatively similar to previously reported rates [24,25,26]. Mortality occurred only in AdCC cases; although survival data were unavailable for some patients, these outcomes are in line with previous studies [26].

Proliferation is a key determinant of tumor growth and biological behavior. Assessing proliferation by Ki-67 expression is considered a simple, rapid, reliable, and widely-used method [4]. NSGs under physiological conditions have occasional isolated Ki-67-positive cells, indicating minimal proliferative activities [27,28]. This is consistent with our findings.

PA is a benign SGT characterized by a slow-growing nature [29]. In the present study, it showed a 3.6% Ki-67 labeling index, which aligns with the results of previous studies (< 5%) [18,29,30,31]. In PA, proliferating cells are equally distributed between peripheral and central regions. This may reflect its lobulated gross architecture. Furthermore, PA is surrounded by either a complete or partial fibrous capsule [32]. Lai et al. indicated that lower Ki-67 expression was more common in highly-cellular PAs and mentioned that such cellular PAs were more frequently associated with incomplete capsulation [18]. Furthermore, the predominant Ki-67 immunopositivity was localized mainly within the epithelial component (cellular areas). This finding aligns with observations by Raja et al., who reported that the cellular type of PA exhibited higher Ki-67 expression than the stroma-rich type [33]. Recently, in a meta-analysis review, De Rosa et al. also confirmed that PA with greater cellularity displays higher Ki-67 expression [34]. Collectively, these observations suggest that epithelial and myoepithelial cells are the origin of this tumor and retain proliferative capacity. In contrast, the mesenchymal-like stromal cells, although derived from myoepithelial cells, appear to be mature and more terminally differentiated, with a greater functional activity than proliferation [32,35]. In our study, WT showed 7% Ki-67 expression, falling within the low-score range. In agreement with the results of Faur et al., who reported that 28.6% of WT cases demonstrated a PI above 5% [36]. In contrast, other studies reported either negative expression or very low PI levels (<5%) [5,37,38].

This variation may be attributed to differences in sample size among studies, tumor size and duration, and patient age [39]. The even spatial distribution of Ki-67 expression observed across WT can be explained similarly to PA (well-capsulated and lobulated growth), as no previous study has evaluated the spatial distribution of proliferation in benign SGTs. Interestingly, Ki-67 expression was higher in basal oncocytes. These results are consistent with previous studies [37,38,40], and Kuzenko et al. considered it as an “active proliferating cyst” [40]. This expression may be influenced by their proximity to lymphoid stroma [41], which is suggested to be directly regulated by basal cells [37]. On the other hand, luminal cells have been suggested to be involved in secretory functions, as cytokeratin-7 and cytokeratin-19 were demonstrated in the columnar cells of WT [42].

Regarding malignant tumors, the present study found higher Ki-67 expression than in benign tumors, consistent with their more aggressive biological behavior [6]. This result aligns with previous studies where both MEC and AdCC had higher PIs than benign tumors [6,43]. In MEC, Ki-67 activity was positively correlated with histological grade. High-grade MECs exhibited higher proliferative activity than low- and intermediate-grade tumors. This pattern aligns with the established grading system, in which high-grade MECs exhibit greater cellularity and invasive potential [44,45]. AdCC exhibited the highest Ki-67 immunostaining. This elevated proliferation activity was particularly evident in a solid histological pattern, which is known to be associated with a poorer prognosis [46]. These findings are consistent with previous studies [5,47].

Apoptosis (programmed cell death) is a fundamental biological process that maintains tissue homeostasis by eliminating damaged or unwanted cells [48]. Dysregulation of apoptosis is a hallmark of tumorigenesis, therapeutic resistance, and disease progression [49]. Therefore, understanding the apoptotic pathways is essential for identifying the biology of SGTs and novel therapeutic targets [50]. Previous studies confirm that early apoptotic events can be detected through the use of ANX-V (a calcium-dependent extracellular and intracellular protein), which precedes the activation of caspases and other downstream molecules, resulting in programmed cell death (late apoptosis) [7,51,52,53]. ANX-V staining can be visualized using various methods, such as light microscopy or flow cytometry, and can be applied to cell cultures to provide quantitative data; however, it lacks histomorphological localization within tissue [7]. This limitation requires markers that are both quantitative and histologically informative. Meanwhile, evaluating apoptosis by ANX-V in tissue sections has been applied to detect apoptosis in dental and periodontal tissues and breast tumors [13,54]. Nevertheless, it has not been studied in SGTs before. ANX-V, besides its role in cell death, plays key roles in membrane organization (endocytosis and exocytosis) and inflammation [9]. Therefore, it has a different cellular localization [10]. In a study by Sacre and Moss et al., ANX-V showed the most striking response to hyperoxia, with immunoreactivity predominantly in the nuclei; thus, nuclear ANX-V was expressed in more than 80% of oxidatively stressed cells [11]. Manoceau et al. and Kenis et al. demonstrated the co-localization of TUNEL and ANX-V in tissue sections of ischemic myocardium, suggesting that intracellular ANX-V redistribution is associated with apoptotic changes [55,56]. The present study represents the first study to investigate ANX-V in SGTs immunohistochemically, and only nuclear ANX-V was interpreted as being associated with early apoptosis [10,11,55], while cytoplasmic and membranous localization was regarded as reflecting other functional roles, such as calcium signaling, membrane repair, or vesicle trafficking [8,9].

In the present study, nuclear ANX-V in NSG was detected as 14.5% positivity. This weak nuclear (perinuclear) localization may be related to the low level of cellular turnover typically observed in SGs under physiological conditions [57]. Additionally, total ANX-V had a strong expression in NSGs, predominantly in the cytoplasm and membrane of serous acini and ductal cells, whereas mucous acini showed little to no reactivity. This expression within serous acini may contribute to their secretory function. They are characterized by abundant zymogen granules and depend heavily on Ca^2+^-regulated exocytosis [58], a process in which ANX-V may be involved [59]. On the other hand, the strong ANX-V expression was at the apical and interproximal membranes of ductal cells. This pattern may be related to their role in saliva modification and secretion, as ductal cells undergo continuous vesicular transport [9,60]. Additionally, the ANX-V role in membrane stabilization and repair, as reported by previous studies, could explain the membrane staining [61,62]. The preferential basal cytoplasmic localization of ANX-V in the SDs of NSGs may be associated with the abundance of mitochondria concentrated within the basal striations. These ducts are characterized by extensive basal infoldings housing numerous mitochondria, supporting active ion transport [63,64]. Accordingly, the peri-membranous basal localization of ANX-V may reflect its spatial distribution within polarized epithelial cells. Previous studies have demonstrated high ANX-V expression in secretory cells of mucous glands in normal pharyngeal epithelium, suggesting a potential association with epithelial cell specialization [65]. Although ANX-V has been implicated in mitochondrial-associated apoptotic pathways [66], such interpretations remain speculative, as no functional assays were performed in the present study.

Other Annexins, such as ANX-I, were investigated in a study by Al-Ghaban et al., which revealed strong expression in myoepithelial and ductal cells, whereas serous acini exhibited little to no staining [67]. In another study, Cardoso et al. examined ANX-II expression in SG tissues and found that it was lower in NSGs than in malignant SGTs [17]. However, the biological roles of ANX-I and ANX-II differ from those of ANX-V, as ANX-I is identified as an inhibitor of phospholipase A2, which can act as a proinflammatory and anti-inflammatory regulator [68], while ANX-II plays roles in cell differentiation, angiogenesis, and extracellular matrix degradation [17].

Regarding the benign tumors among all study groups, PA exhibited the greatest nuclear ANX-V values, which may be related to the increased susceptibility of mesenchymal (stromal) cells to metabolic cellular stress during the differentiation process that involves significant cytoskeletal remodeling, as Yamaguchi et al. demonstrated the expression of nitric oxide and heat shock proteins in these cells [69,70], and ANX-V has been reported to participate in cytoskeleton dynamics [71]. Moreover, the total ANX-V expression in PA had an expression of 53%, which could be related to the presence of bone and cartilage within the tumor, as ANX-V has been recognized to play an active role in the osteogenesis and mineralization of chondrocytes [72,73]. Similar ANX-V positivity was observed in both the peripheral and central areas of PA; this finding is consistent with Ki-67 expression in these regions. This pattern may reflect a relatively homogeneous biological behavior of PA [32].

In WT, nuclear ANX-V expression was mainly restricted to the basal layer. This may be influenced by interaction with the lymphoid stroma and the distinct profile of basal cells [40]. Supporting this, Mandic et al. reported the predominant expression of GAPDH (an enzyme associated with damaged mitochondria and involved in oxidative signaling) in the basal epithelial cells of WT, suggesting that basal oncocytes may experience elevated oxidative stress, which may explain the observed nuclear ANX-V expression [11,74,75]. Previous studies have also reported altered ANX-V nuclear localization in tumor cells of colon adenocarcinoma in response to apoptotic stimuli [10]. The total ANX-V was moderately expressed and mainly located at the luminal membrane of oncocytes, as they are involved in the release of secretory materials into cystic spaces [40]. The central area of the tumor exhibited slightly higher ANX-V expression compared to the peripheral area. This result could be influenced by the counting methods used in our study, as only the epithelial component was considered, while the lymphoid stroma was excluded from the evaluation.

In evaluating malignant tumors, both MEC and AdCC showed weak nuclear ANX-V expression. This observation may suggest differences in the biological behavior of SGTs. Previous studies have reported the increased expression of anti-apoptotic proteins and reduced caspase-3 activity in these tumors, suggesting alterations in apoptotic signaling pathways [76,77]. ANX-V was primarily localized in the cell membrane of low-grade MECs and the tubular pattern of AdCCs. This observation may be associated with the proposed roles of ANX-V in membrane-related processes, particularly in tumors that exhibit microcystic spaces and secretory activity [1,8,9]. In addition, membrane localization of ANX-V in head and neck squamous cell carcinomas has been suggested to correlate with cellular mobility [65].

The lack of correlation within individual tumor types indicates that Ki-67 and nuclear ANX-V expression are likely regulated independently in both benign and malignant SGTs. The observed overall correlation appears to reflect inter-tumor differences rather than a biologically meaningful association within SGTs. The relationship between ANX-V expression and tumor behavior appears variable across tumor types; for example, both ANX-V and nuclear ANX-V have been reported to increase with malignancy in hepatocellular carcinoma, whereas an inverse relationship has been observed in thyroid follicular carcinoma [78,79,80].

The net change is expressed as the Ki-67-to-nuclear ANX-V ratio (Ki-67/nuclear ANX-V), a relative index of proliferation in relation to ANX-V expression. However, given that nuclear ANX-V staining was used in this study as an indicator of early apoptosis, it should be noted that the absence of established apoptotic markers (e.g., cleaved caspase-3, TUNEL) limits the ability to validate the apoptotic significance of ANX-V expression. Within this limitation, a higher ratio therefore suggests proliferative dominance, whereas a lower ratio may indicate the opposite trend. In the present study, our findings of an elevated Ki-67/nuclear ANX-V ratio in malignant tumors compared with benign neoplasms and NSG tissue suggest that malignant SGTs exhibit enhanced proliferation with reduced apoptotic signaling. In uterine cervical carcinoma, increased ANX-V has been reported to be associated with reduced cellular proliferative activity [81]. This observation may further support an association between ANX-V and variation in proliferative behavior across tumor types. Although no significant association was found between Ki-67 and ANX-V and tumor recurrence, a significant correlation between these markers and survival outcome was observed. However, these findings should be considered preliminary and do not allow definitive conclusions regarding prognostic significance, given the limited follow-up data.

This study is subject to several limitations. The retrospective design restricts control over case selection and data completeness. In addition, the relatively small sample sizes in certain subgroups may have limited the statistical power of subgroup analyses. Furthermore, the absence of established apoptotic markers limits the ability to validate nuclear ANX-V as an apoptotic marker. Despite these limitations, these findings suggest that the combined assessment of Ki-67 and ANX-V may serve as a useful tool for distinguishing tumor behavior in SGTs. However, future studies with larger sample sizes, a prospective design, and the inclusion of established apoptotic markers are recommended to validate and expand these results.

## 5. Conclusions

This study demonstrated significant differences in Ki-67 and ANX-V expression among NSG, benign, and malignant tumors. Ki-67 expression tended to increase with malignancy and tumor grade, although its generally low expression may reflect the inherently slow-growing nature of SGTs. In contrast, ANX-V showed higher expression in NSG tissue and benign tumors, and its distinct cellular localization may indicate diverse functional roles in physiological processes and tumor biology. Within individual tumor types, Ki-67 and nuclear ANX-V exhibited no significant correlation, suggesting that they are independently regulated in SGTs.

The observed correlations between these markers and outcome data were limited and should be considered preliminary, providing exploratory insight into the identification of biomarkers that may reflect the tumor growth dynamics. These findings support further clinicopathological evaluation of the combined expression of Ki-67 and ANX-V.

## Figures and Tables

**Table 1:** Demographic and clinical characteristics of the studied salivary gland tumor (SGT) samples: pleomorphic adenoma (PA), Warthin tumor (WT), mucoepidermoid carcinoma (MEC), and adenoid cystic carcinoma (AdCC).

Variables		Total	PA	WT	MEC	AdCC	*p*-Value
		No. (%)	No. (%)	No. (%)	No. (%)	No. (%)	
Age		48 ± 14	39.5 ± 18	54.6 ± 18	51 ± 11.6	47 ± 12.7	0.086
Sex	Male	25 (62.5%)	3 (30%)	9 (90%)	6 (60%)	7 (70%)	0.046
	Female	15 (37.5%)	7 (70%)	1 (10%)	4 (40%)	3 (30%)	
Site	Parotid	17 (43%)	5 (50%)	10 (100%)	2 (20%)	0	<0.001
	Submandibular	5 (12%)	2 (20%)	0	1 (10%)	2 (20%)	
	Minor	18 (45%)	3 (30%)	0	7 (70%)	8 (80%)	
Size (cm) *		3.25 ± 2	2.49 ± 1.35	3.73 ± 1.35	3.1 ± 2.9	3.8 ± 2.2	0.206
Recurrence status	Primary	33 (82.5%)	9 (90%)	10 (100%)	8 (80%)	6 (60%)	0.109
	Recurrent	7 (17.5%)	1 (10%)	0	2 (20%)	4 (40%)	
Survival outcome **	Alive	28 (93%)	9 (100%)	10 (100%)	5 (100%)	4 (67%)	0.036
	Dead	2 (7%)	0	0	0	2 (33%)	

* The sizes of two AdCC cases were unavailable. ** The outcome of 10 cases was unknown.

**Table 2:** Expression of Ki-67 and ANX-V in salivary gland neoplasm (%).

Tumor Type	Parameters		No.	Ki-67 (%)	ANX-V (%)
PA	Spatial distribution	Periphery	10	3.2	54
		Center		3.4	53
	Cellular distribution *	Epithelial component	10	68	20
		Mesenchymal component		32	80
WT	Spatial distribution	Periphery	10	7	41
		Center		8	45
	Cellular distribution *	Luminal	10	14	66
		Basal		86	34
MEC	Histologic grade	Low	3	6	55
		Intermediate	4	10.75	37.25
		High	3	18	30
AdCC	Histologic pattern **	Cribriform	3	17.5	23
		Tubular	3	15.5	33
		Solid	4	37	17

* Parameters were calculated relative to the total number of positive cells only. ** Some cases exhibited a mixed histological pattern. Cases were assigned to groups based on their most predominant pattern.

**Table 3:** Correlation of Ki-67 and ANX-V expression with recurrence status and survival outcome in SGTs.

Parameters		Ki-67 Mean ± SD (%)	Correlation (r, *p*-Value)	ANX-V Mean ± SD (%)	Correlation (r, *p*-Value)
Recurrence status	Primary	10.4 ± 1.7	r = 0.186 *p* = 0.251	41.7 ± 3.5	r = −0.198 *p* = 0.222
	Recurrence	16.5 ± 5.3		31 ± 9	
Survival outcome	Alive	8.97 ± 1.7	r = 0.402 *p* = 0.028	42.8 ± 3.7	r = −0.421 *p* = 0.021
	Dead	34.7 ± 8.3		7.5 ± 3.5	

## Data Availability

The data supporting the findings of this study are not publicly available due to ethical restrictions, as individual informed consent was not obtained. The data are available from the corresponding author upon request and subject to institutional and ethical approval.

## Supplementary Materials

The following supporting information can be downloaded at: https://www.mdpi.com/article/10.3390/cimb48040387/s1.

## Abbreviations

The following abbreviations are used in this manuscript:

AdCC	Adenoid cystic carcinoma
ANX-I	Annexin I
ANX-II	Annexin II
ANX-V	Annexin V
FFPE	Formalin-fixed, paraffin-embedded
ICC	Interclass correlation coefficient
IHC	Immunohistochemistry
MEC	Mucoepidermoid carcinoma
NSG(s)	Normal salivary gland(s)
PA	Pleomorphic adenoma
PI	Proliferation index
SD(s)	Striated ducts
SG(s)	Salivary gland(s)
SGT(s)	Salivary gland tumor(s)
WT	Warthin tumor
