# Supplementary material for: Role of Ki-67 and Annexin V in the Biological Behavior of Salivary Gland Tumors: Insights into Proliferation and Apoptosis
Source: Curr Issues Mol Biol. 2026 Apr 10;48(4):387. doi: 10.3390/cimb48040387 (PMC13114933; doi:10.3390/cimb48040387)
Supplement: Supplementary file 1 [file cimb-48-00387-s001.zip › Supplementary File S1.pdf]

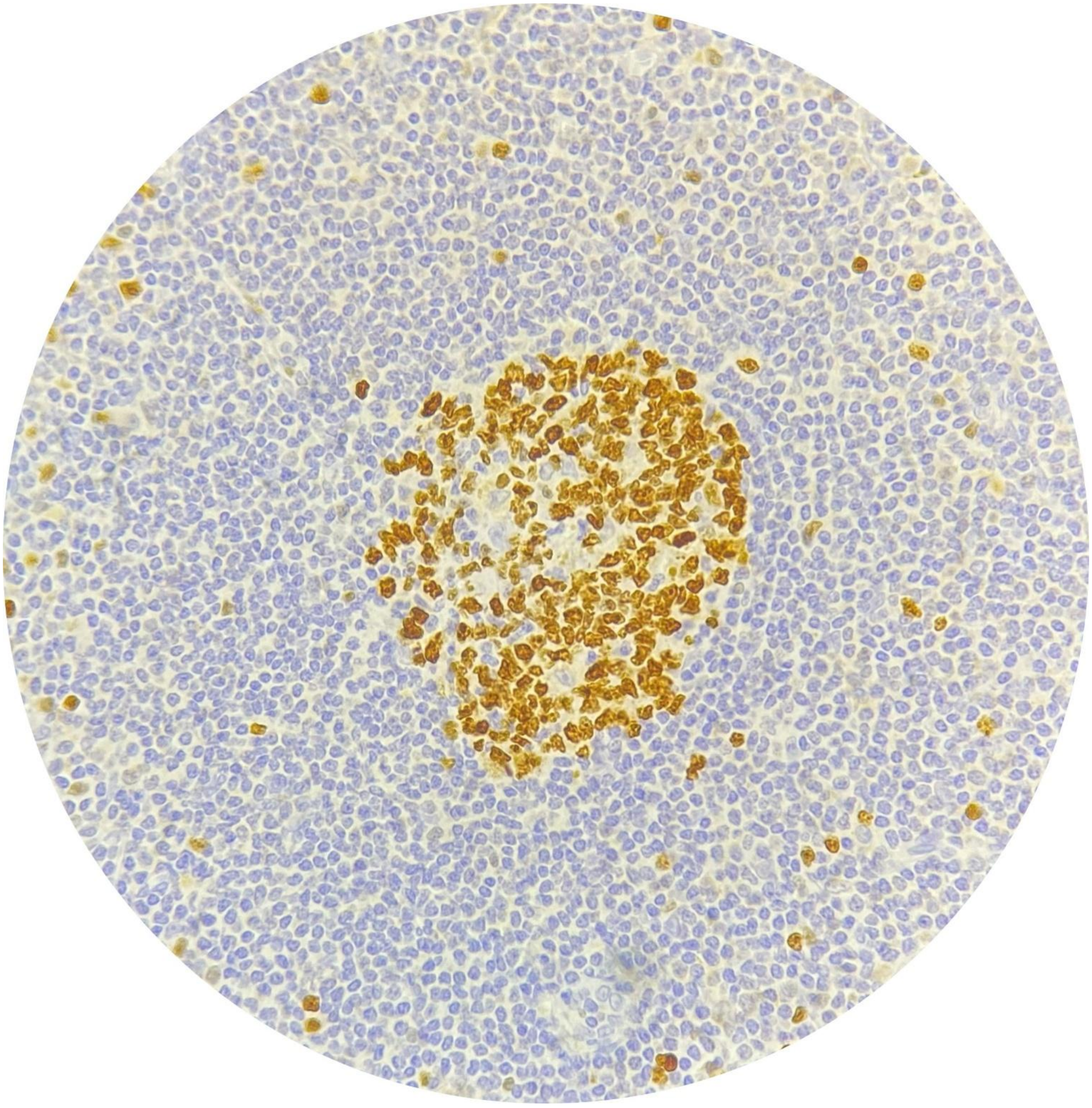

Figure S 1. Positive control showing nuclear Ki-67 staining in human tonsil tissue (IHC, Magnification X400).

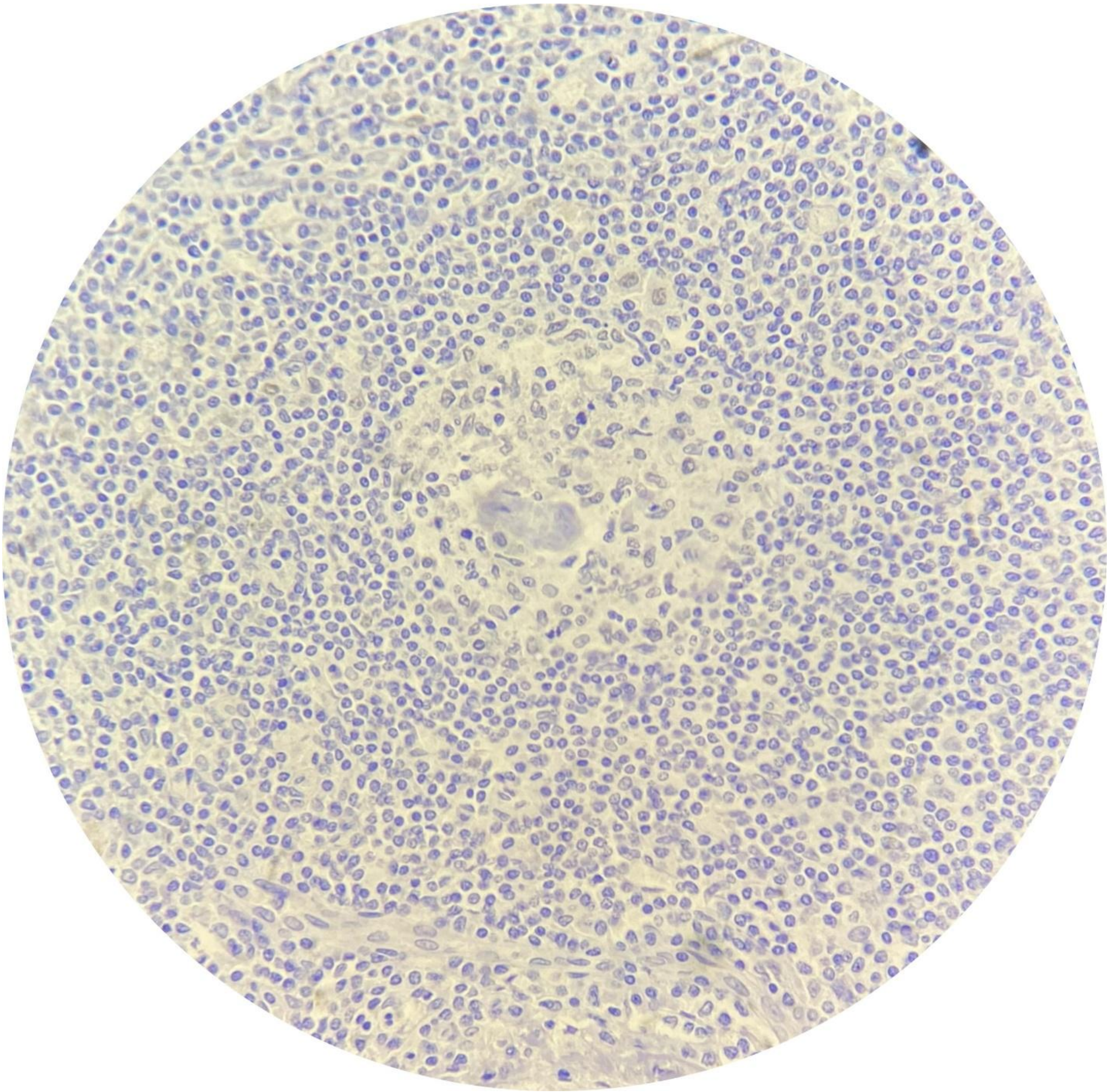

Figure S 2. Negative control showing absence of immunoreactivity of Ki-67 staining in human tonsil tissue (IHC, Magnification X400).

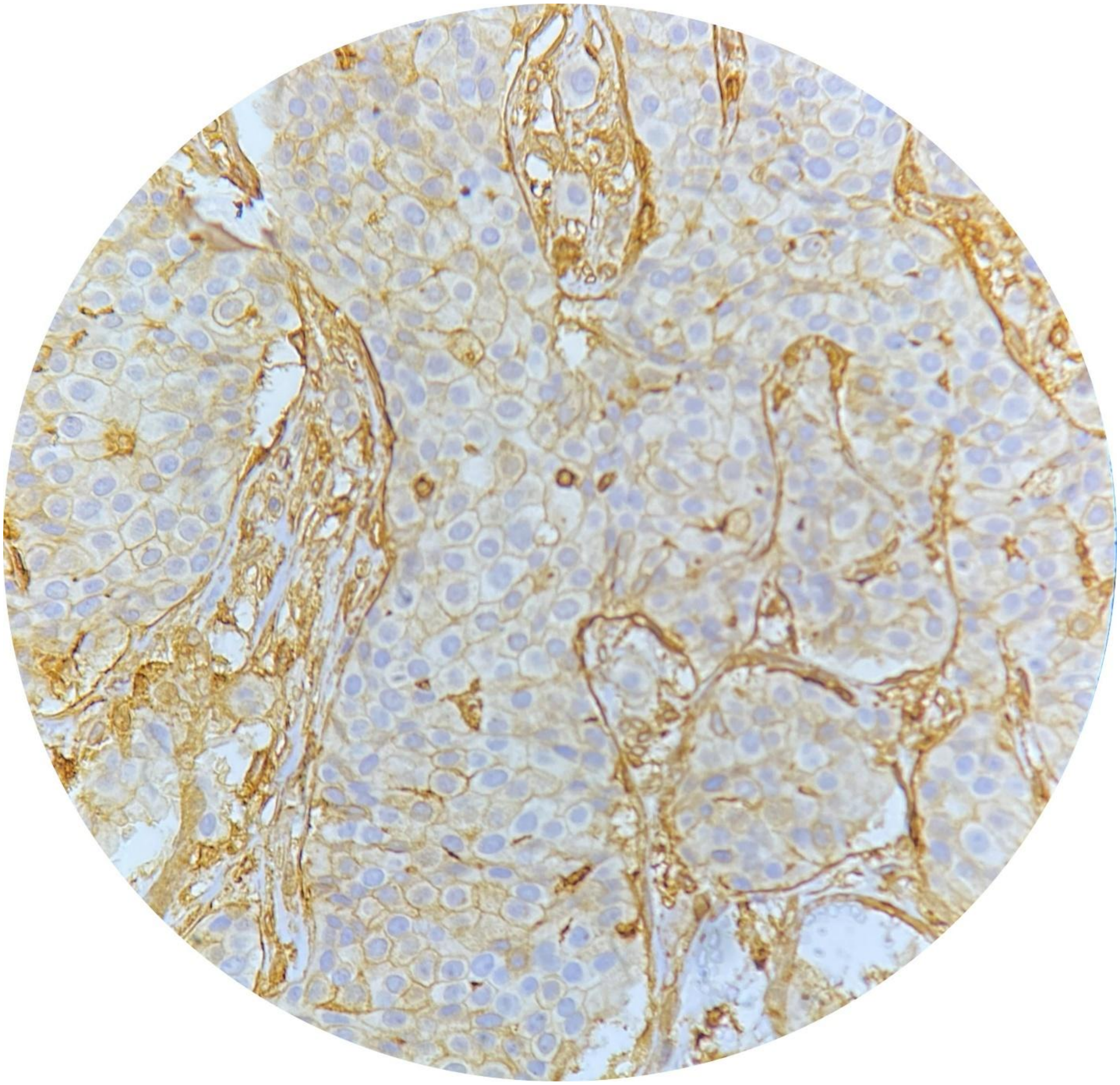

Figure S 3. Positive control showing nuclear, cytoplasmic and membranous Annexin-V staining in breast adenocarcinoma tissue (IHC, Magnification X400).

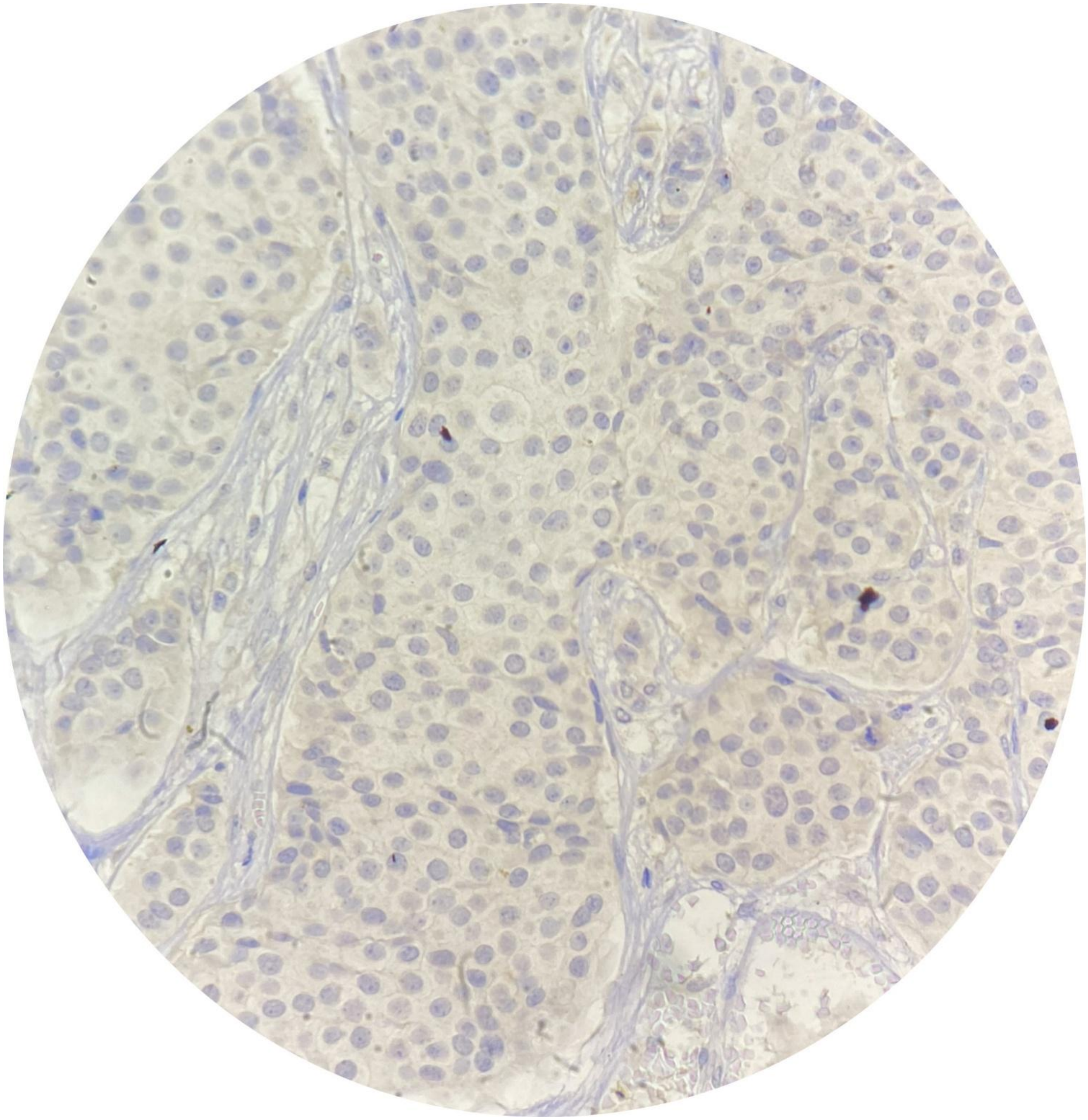

Figure S 4. Negative control showing absence of immunoreactivity of Annexin-V staining in human breast adenocarcinoma (IHC, Magnification X400).
