# Supplementary material for: Role of Ki-67 and Annexin V in the Biological Behavior of Salivary Gland Tumors: Insights into Proliferation and Apoptosis
Source: Curr Issues Mol Biol. 2026 Apr 10;48(4):387. doi: 10.3390/cimb48040387 (PMC13114933; doi:10.3390/cimb48040387)
Supplement: Supplementary file 1 [file cimb-48-00387-s001.zip › Supplementary File S2.pdf]

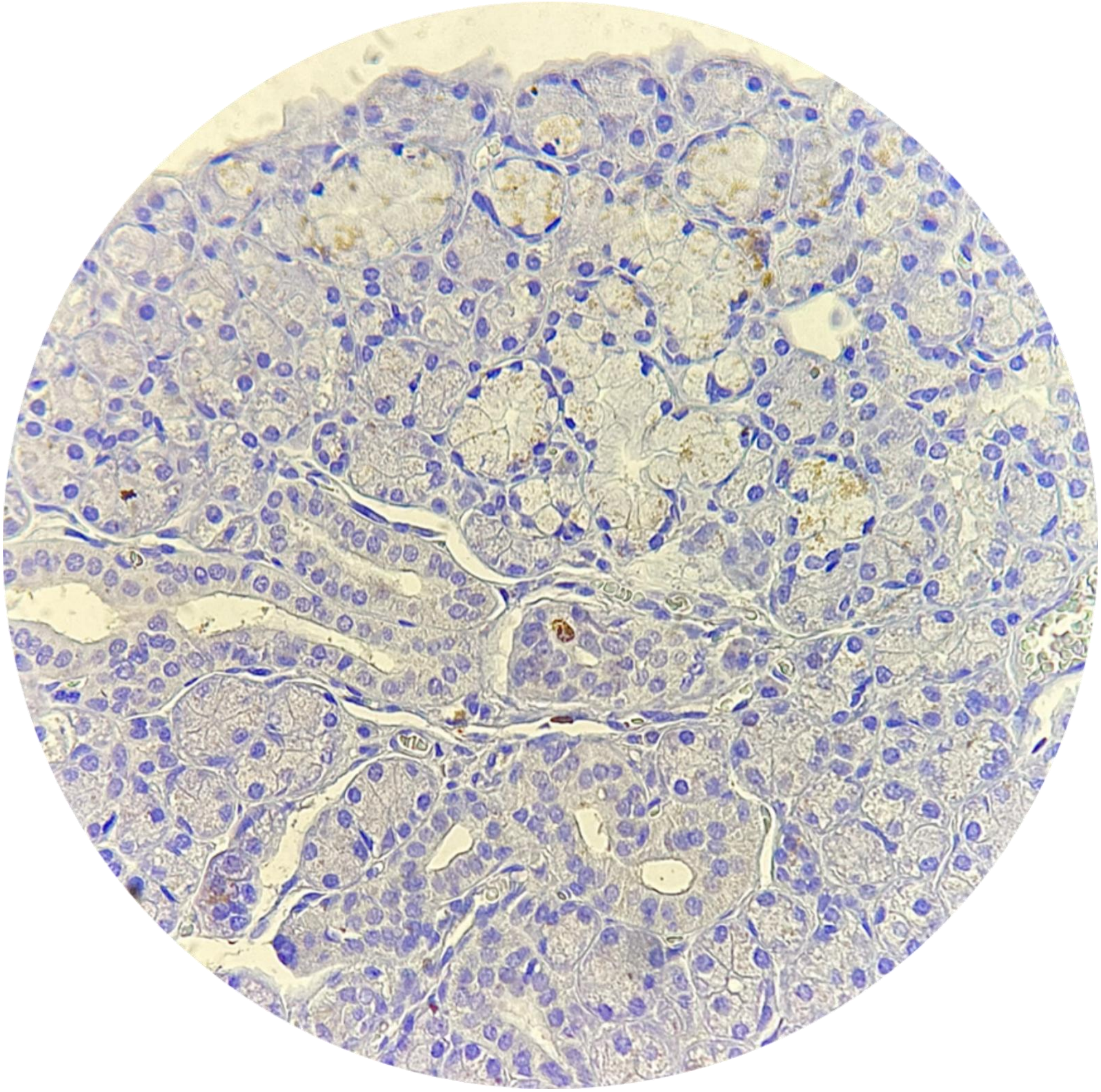

Figure S5. Complete microscopic field of the same NSG section shown in Figure 1. A (IHC, x400).

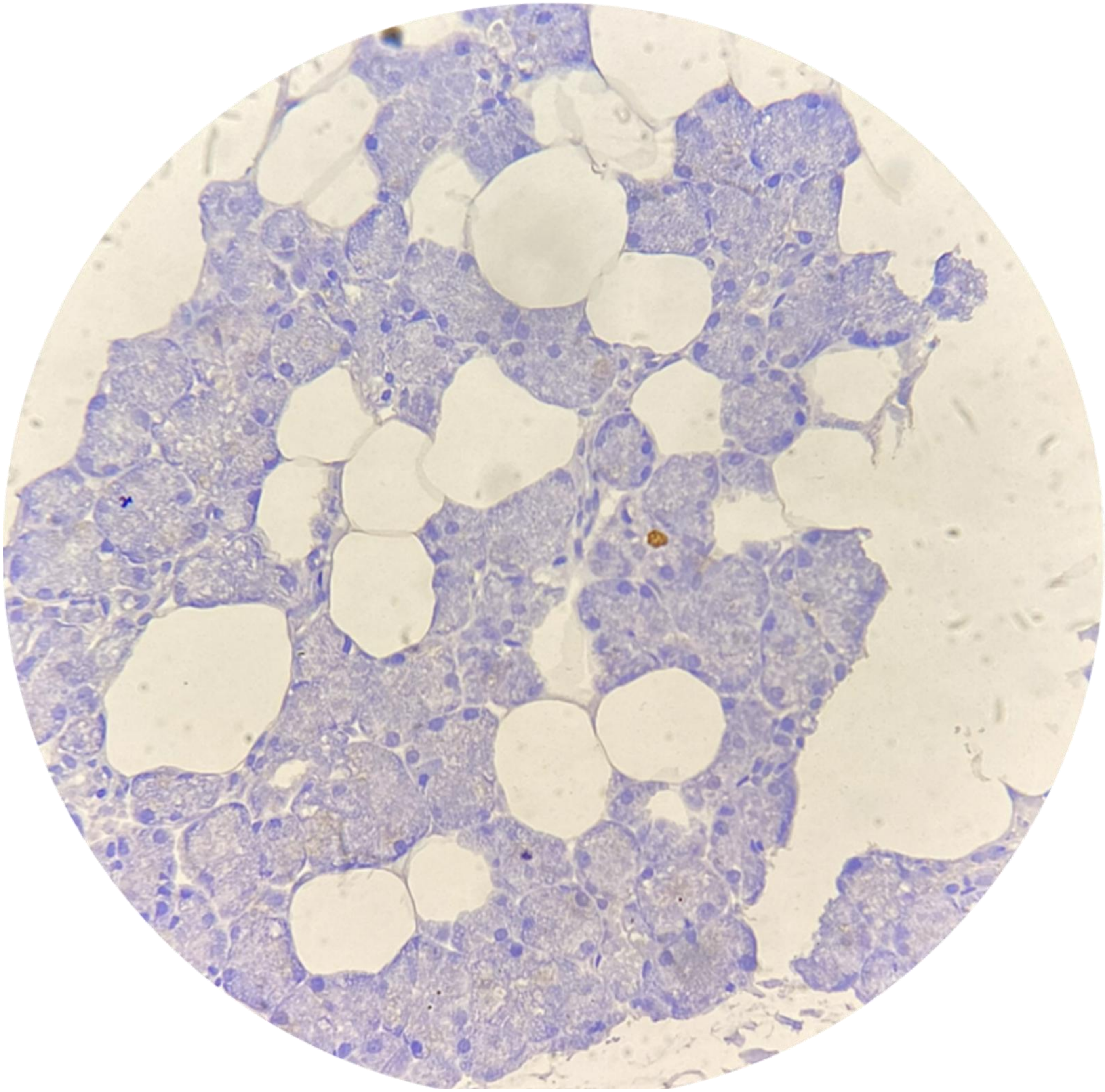

Figure S6. Complete microscopic field of the same NSG section shown in Figure 1. B (IHC, x400).

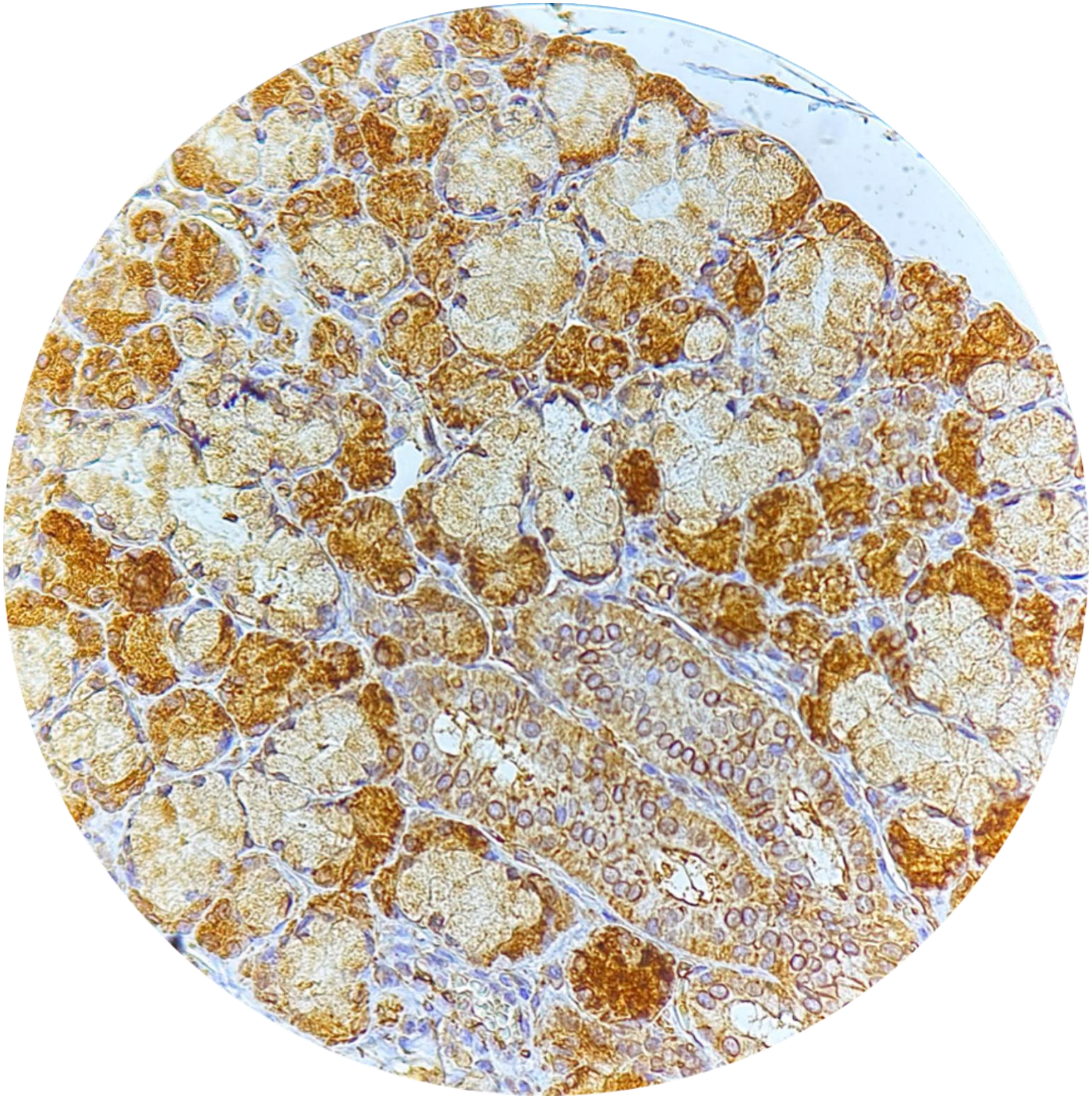

Figure S7. Complete microscopic field of the same NSG section shown in Figure 1. C (IHC, x400).

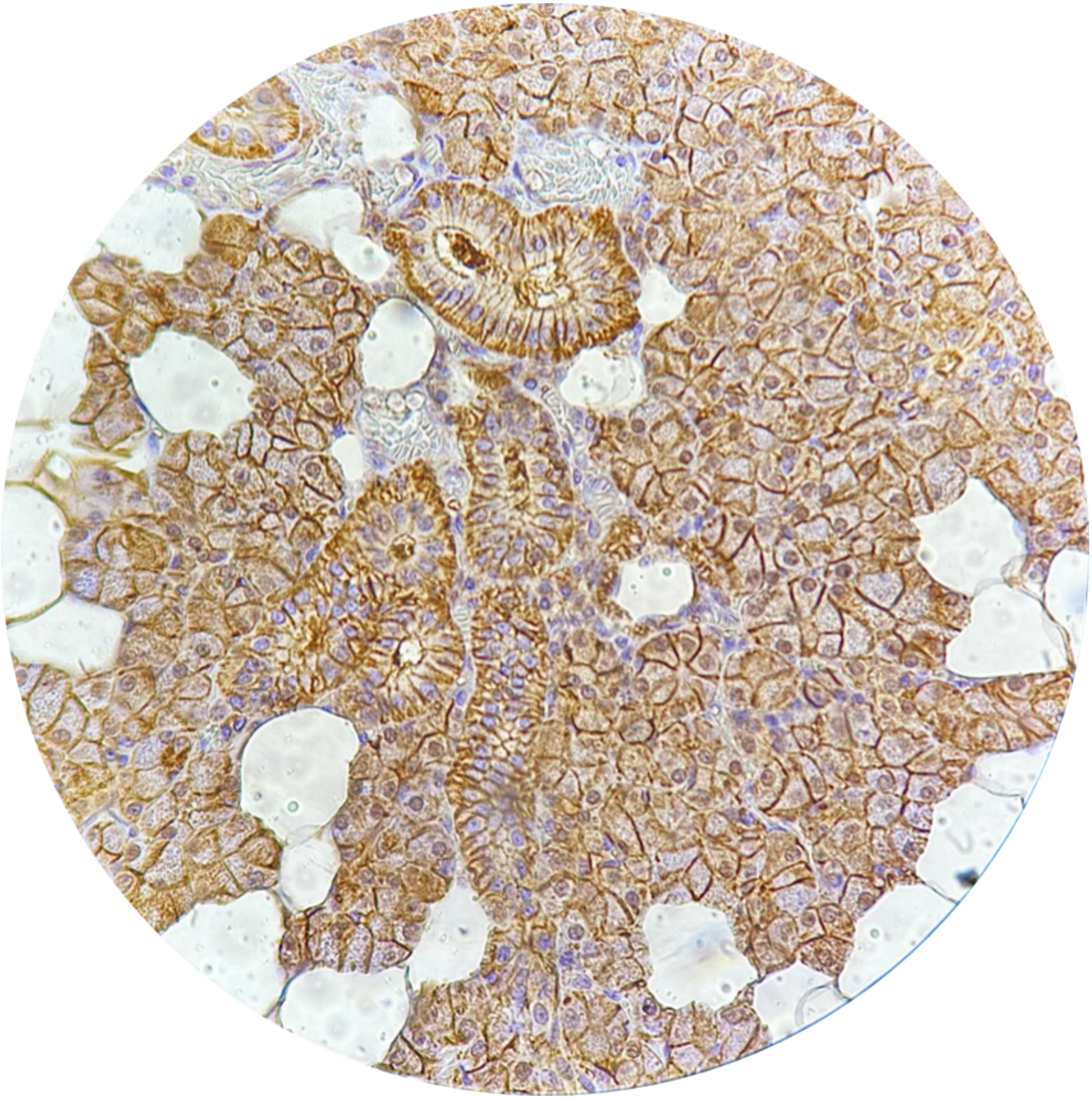

Figure S8. Complete microscopic field of the same NSG section shown in Figure 1. D (IHC, x400).

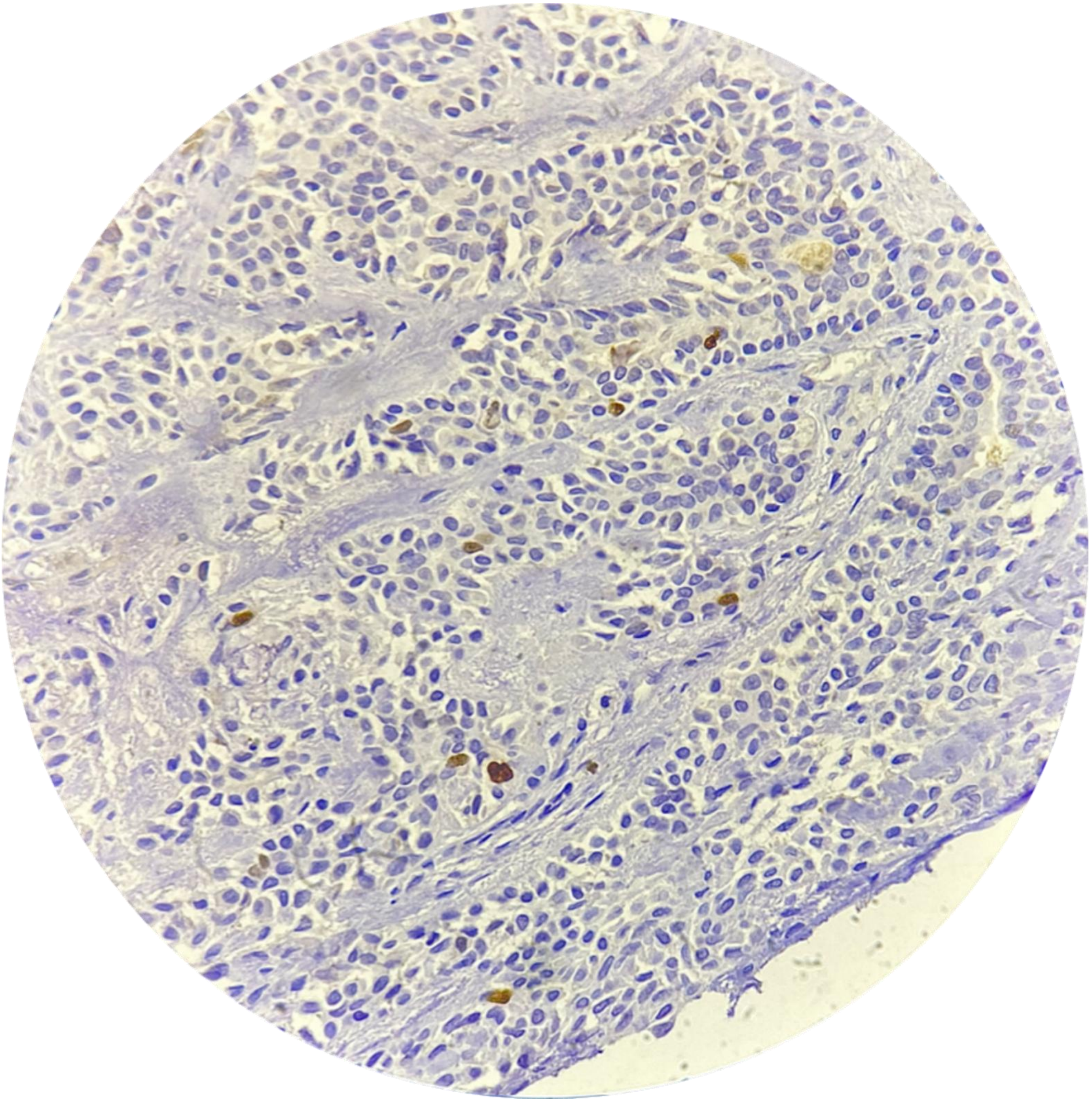

Figure S9. Complete microscopic field of the same PA section shown in Figure 1. E (IHC, x400).

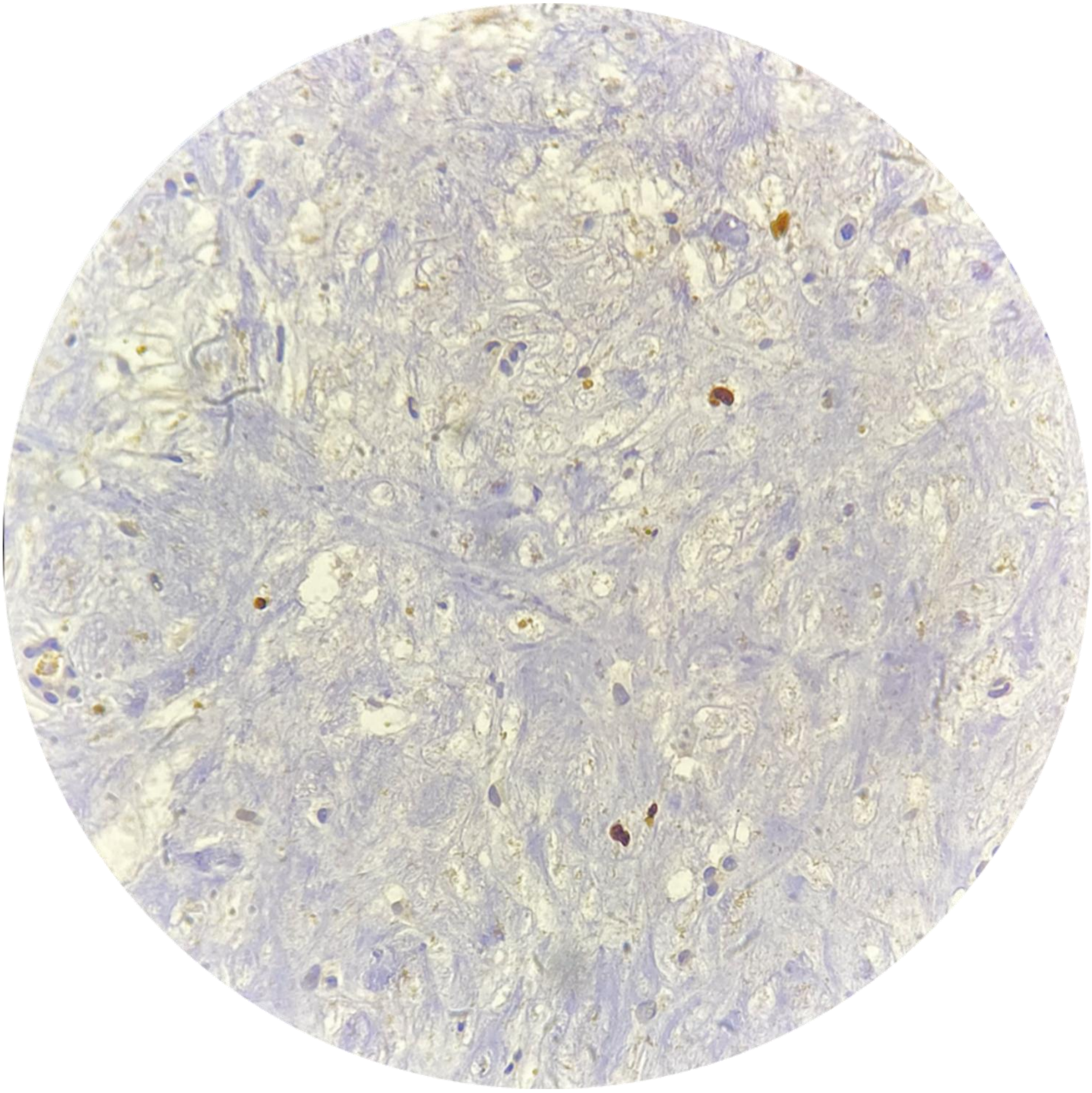

Figure S10. Complete microscopic field of the same PA section shown in Figure 1. F (IHC, x400).

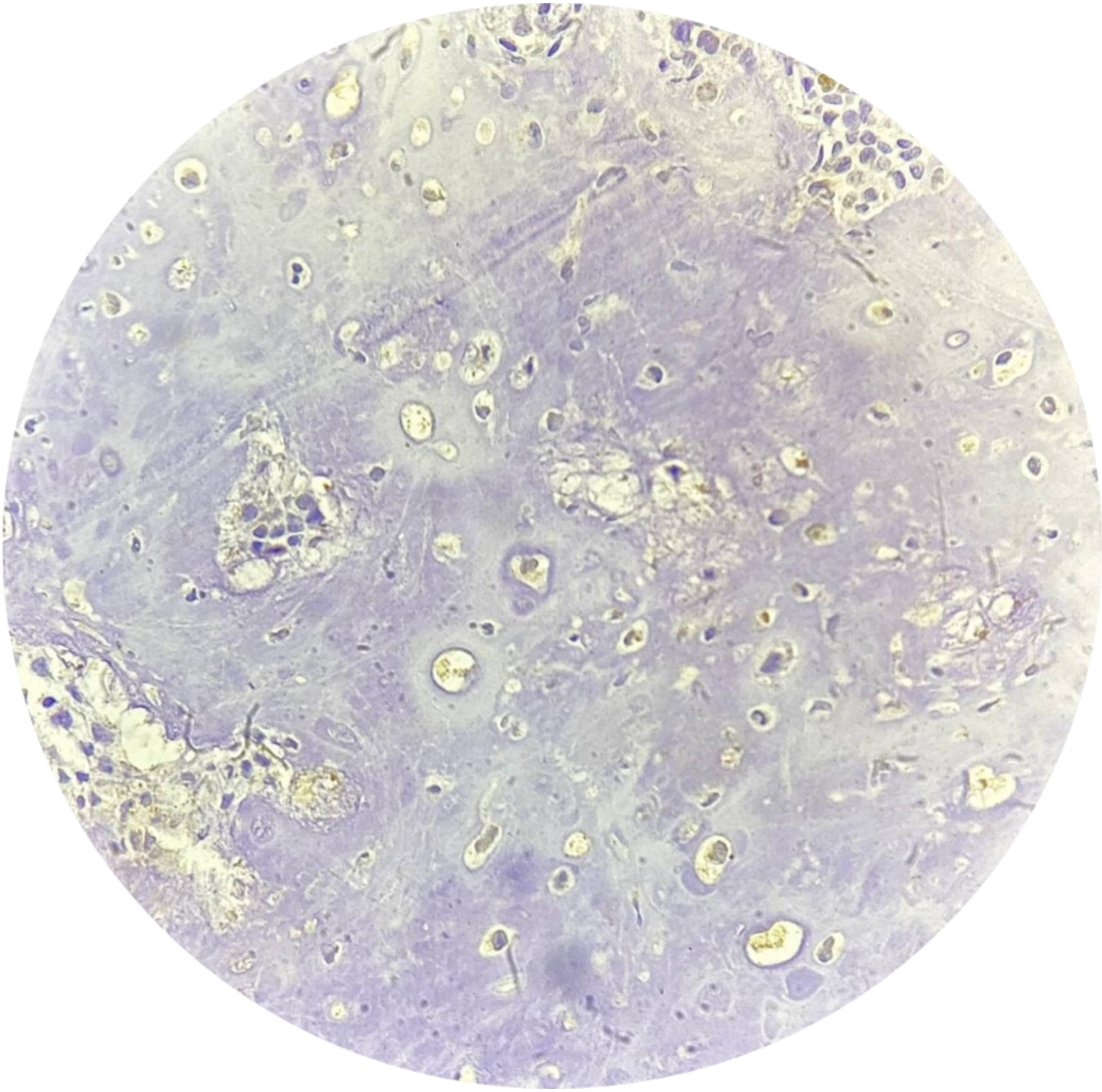

Figure S11. Additional microscopic field of the same PA section shown in Figure 1. F (IHC, x400).

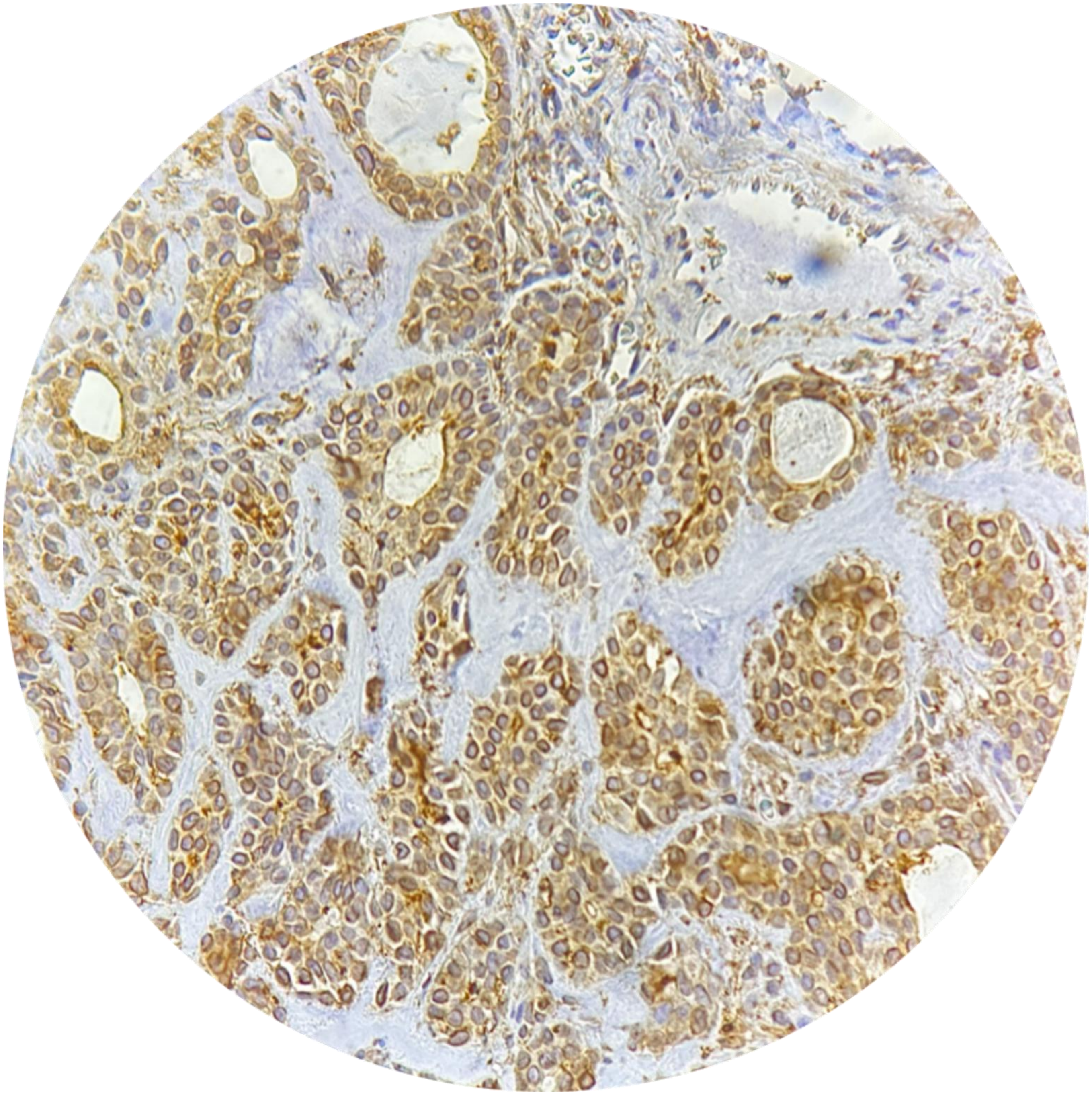

Figure S12. Complete microscopic field of the same PA section shown in Figure 1. G (IHC, x400).

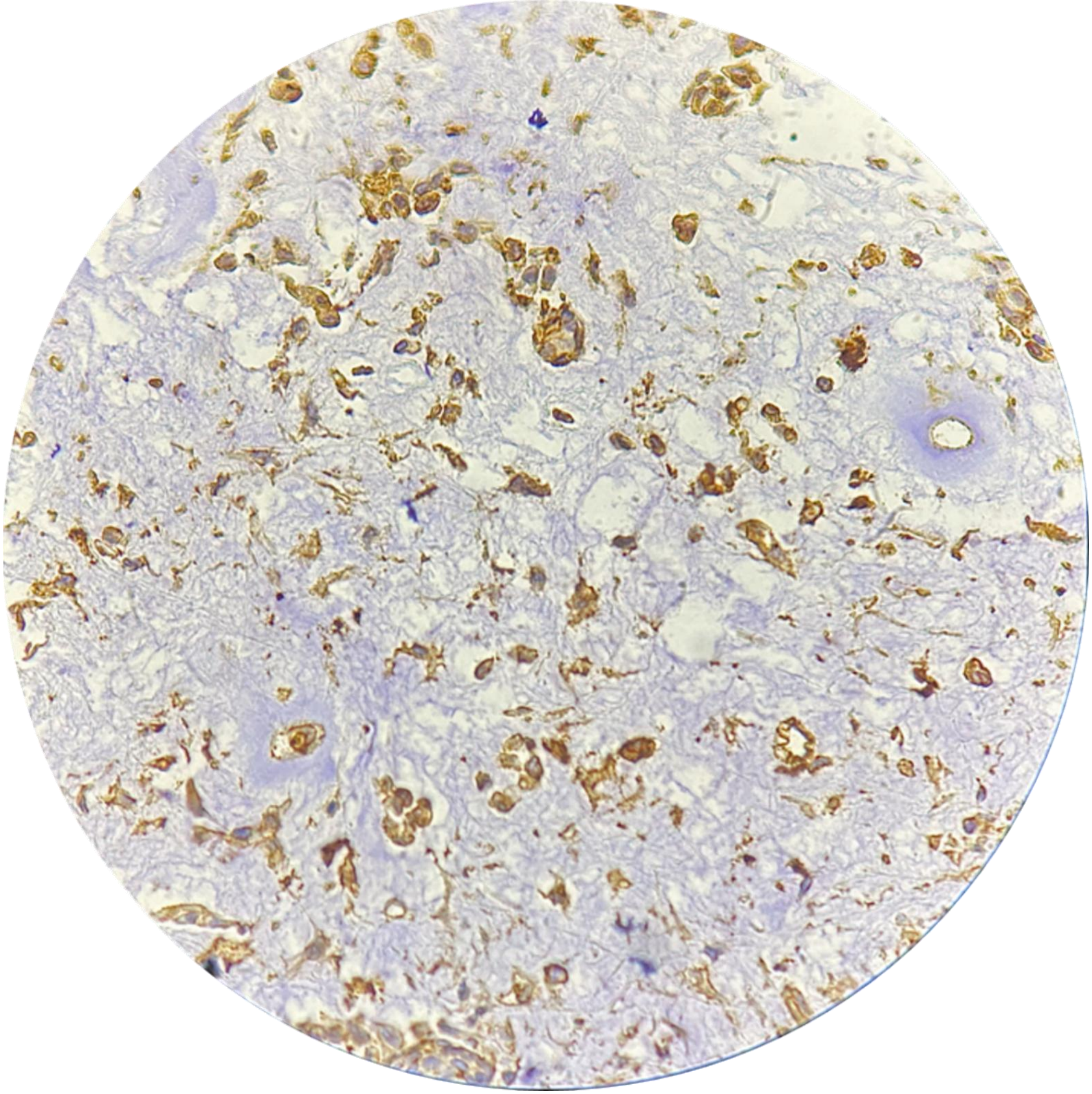

Figure S13. Complete microscopic field of the same PA section shown in Figure 1. H (IHC, x400).

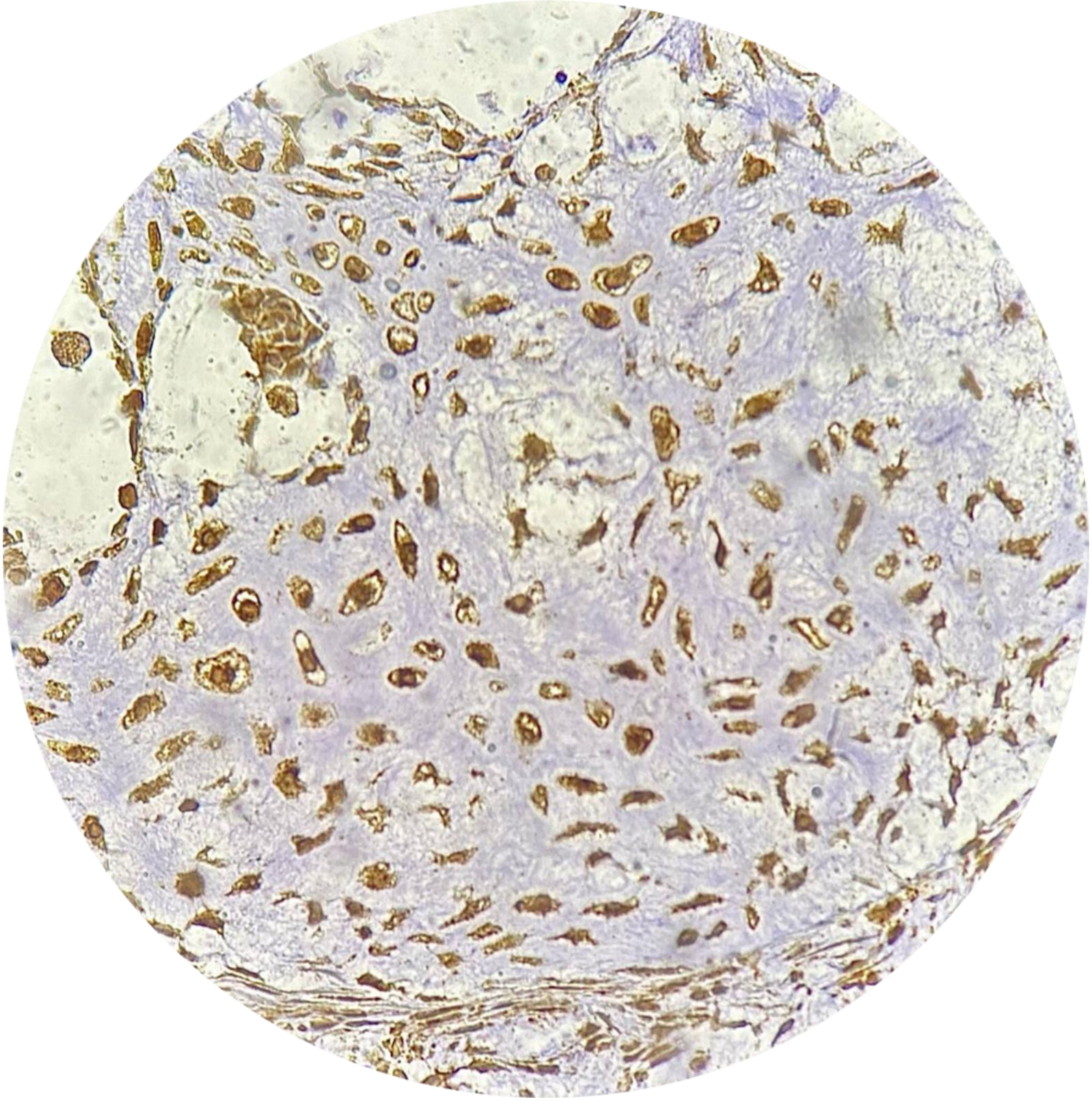

Figure S14. Additional microscopic field of the same PA section shown in Figure 1. H (IHC, x400).

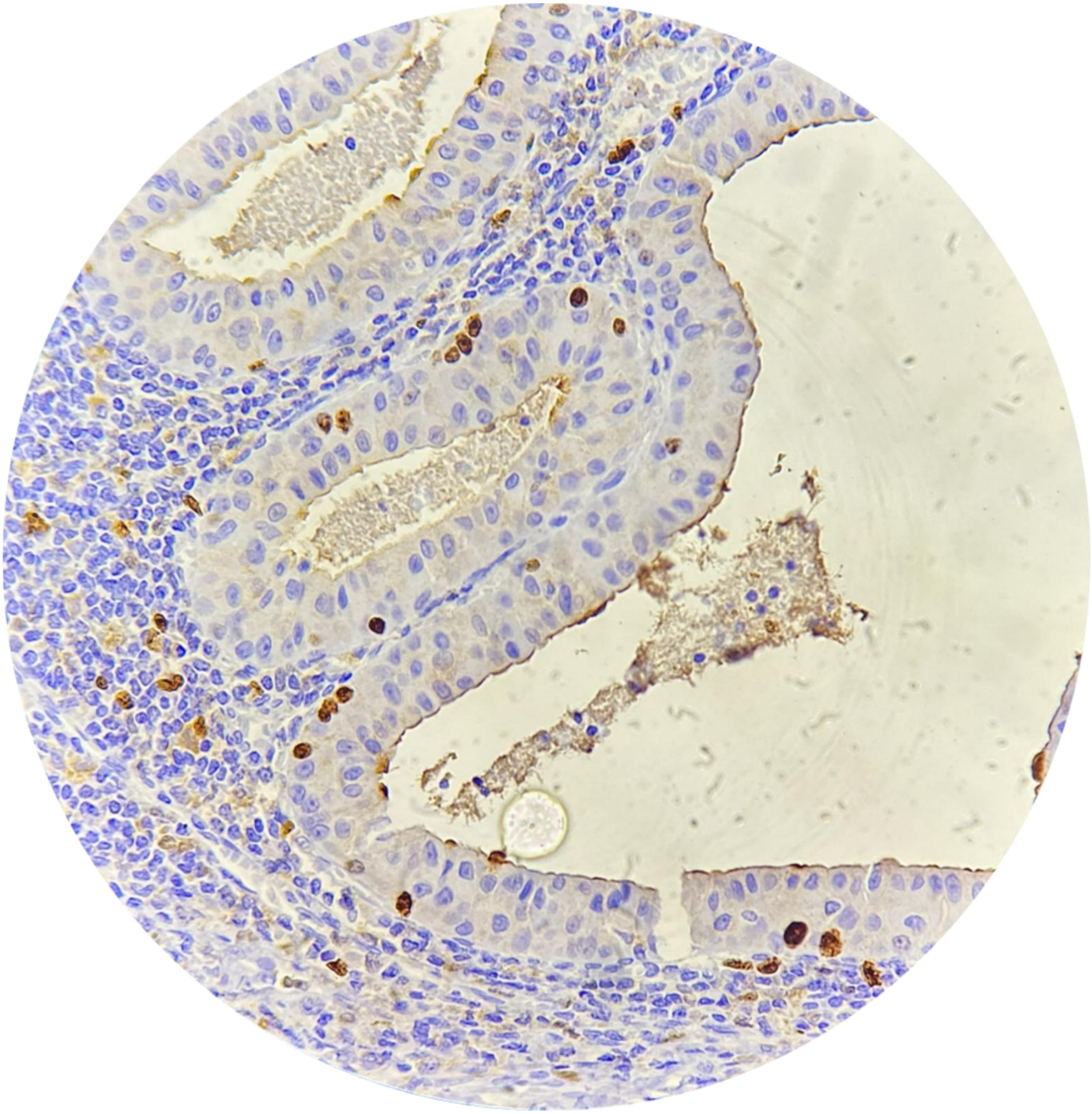

Figure S15. Complete microscopic field of the same WT section shown in Figure 1. I (IHC, x400).

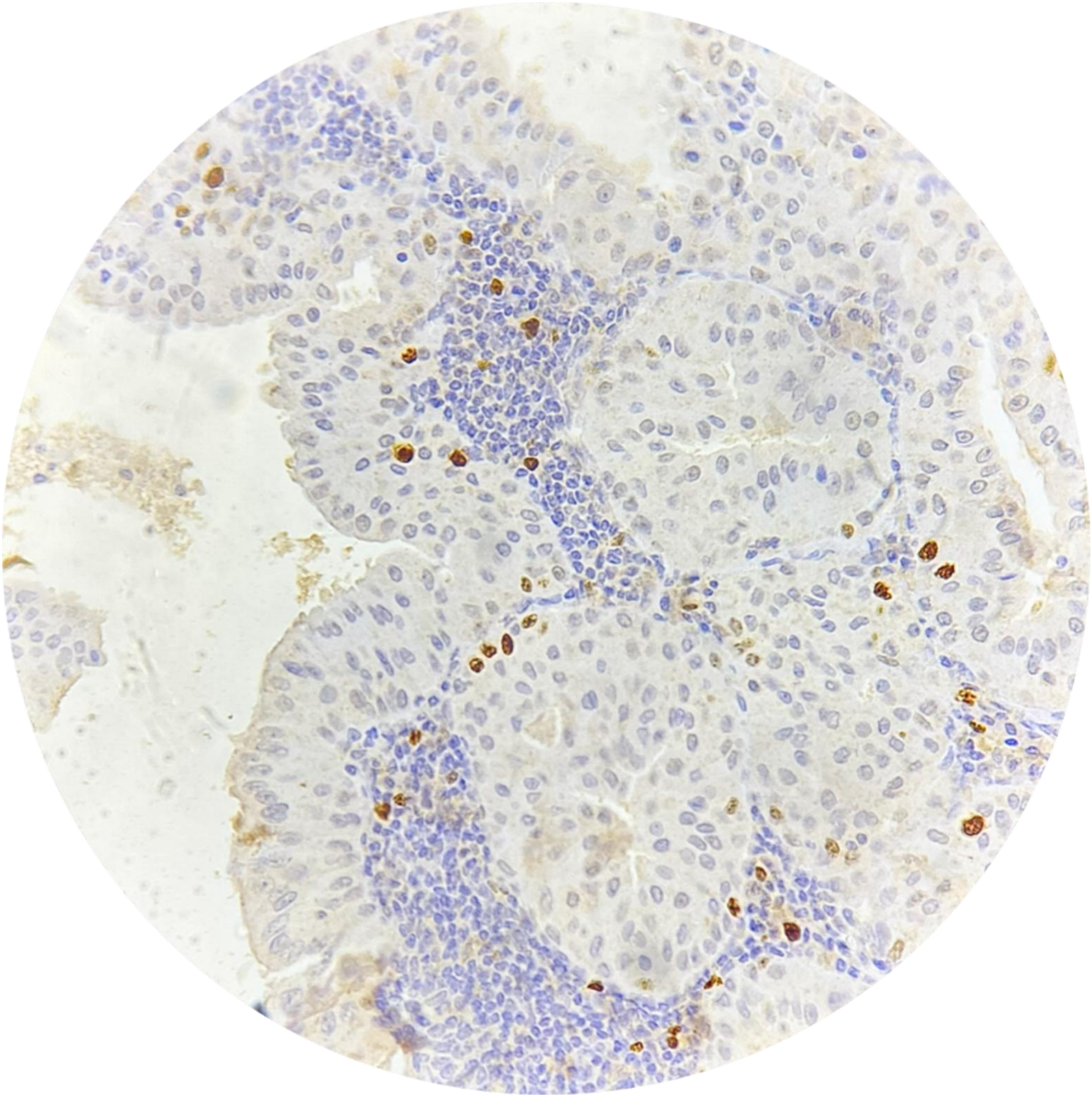

Figure S16. Complete microscopic field of the same WT section shown in Figure 1. J (IHC, x400).

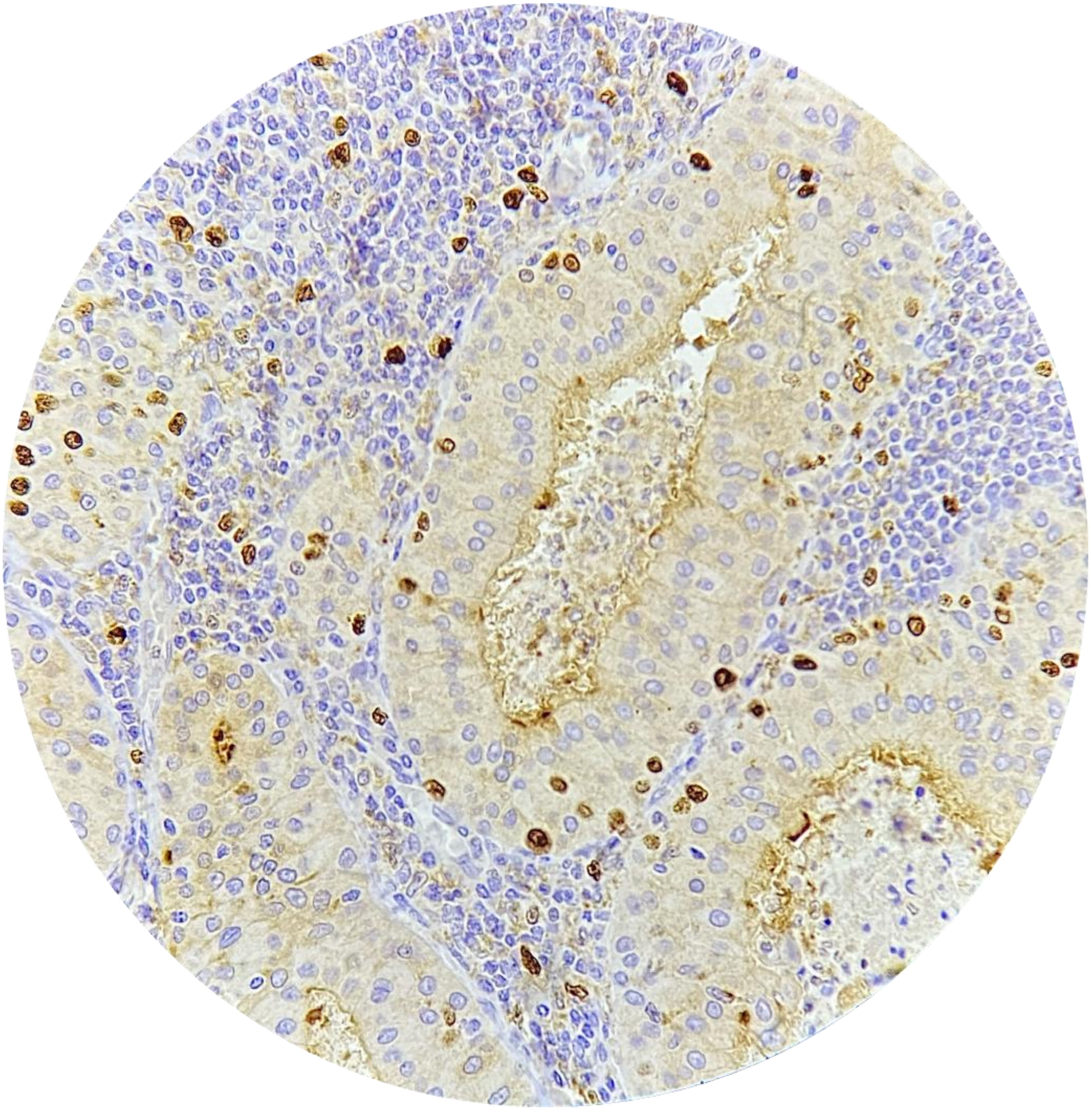

Figure S17. Additional microscopic field of the same WT section shown in Figure 1. J (IHC, x400).

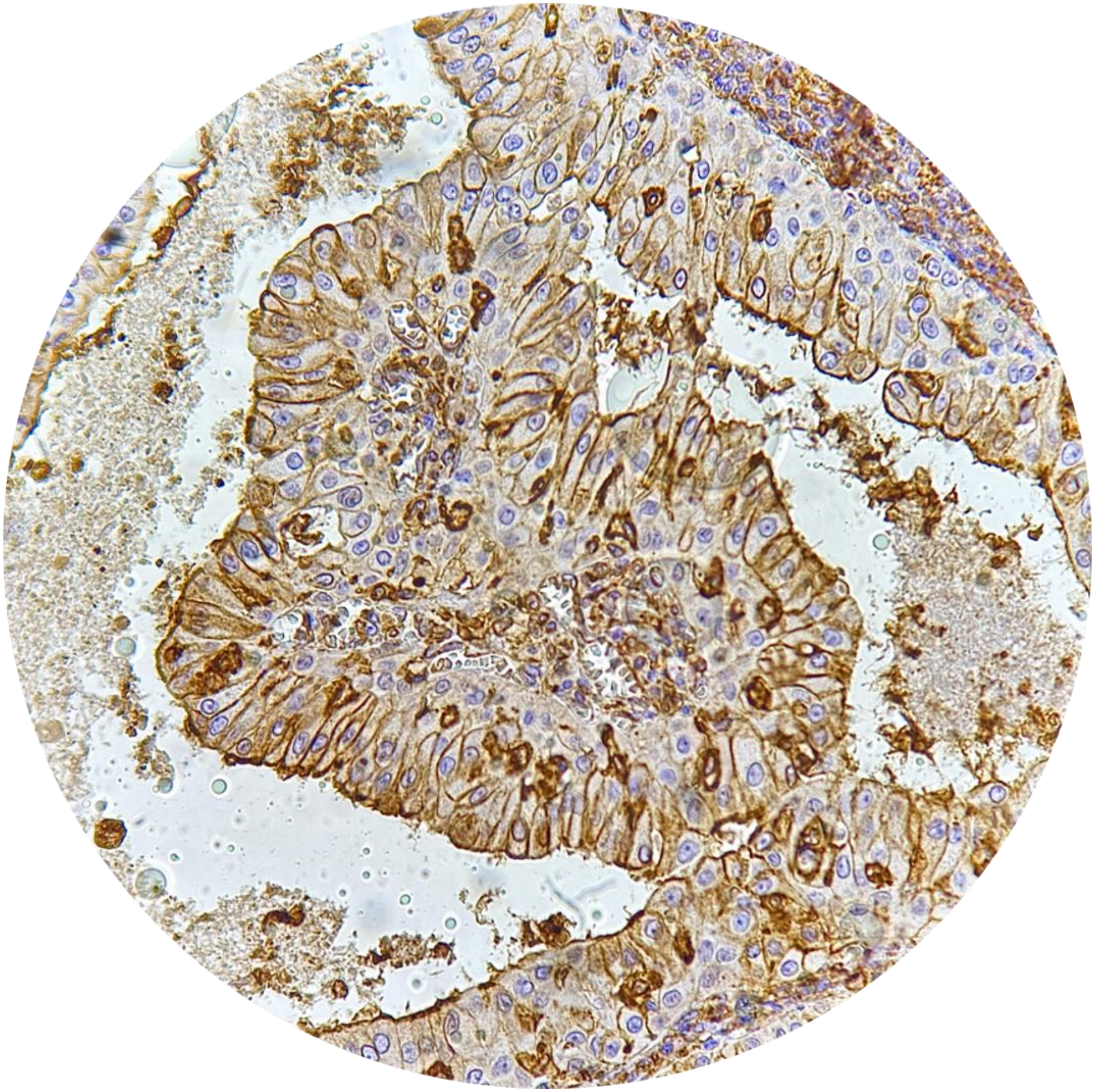

Figure S18. Complete microscopic field of the same WT section shown in Figure 1. K (IHC, x400).

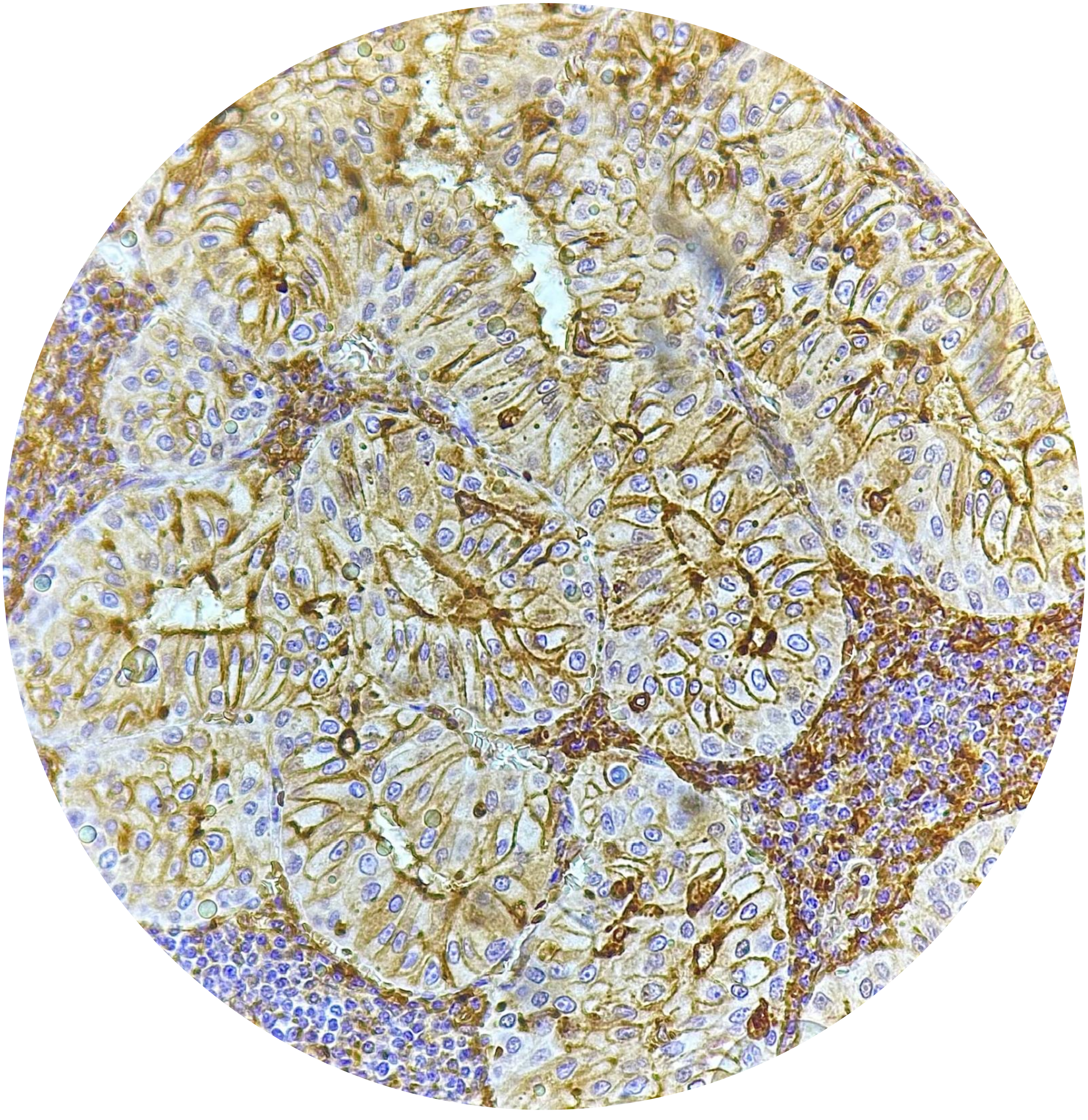

Figure S19. Additional microscopic field of the same WT section shown in Figure 1. K (IHC, x400).

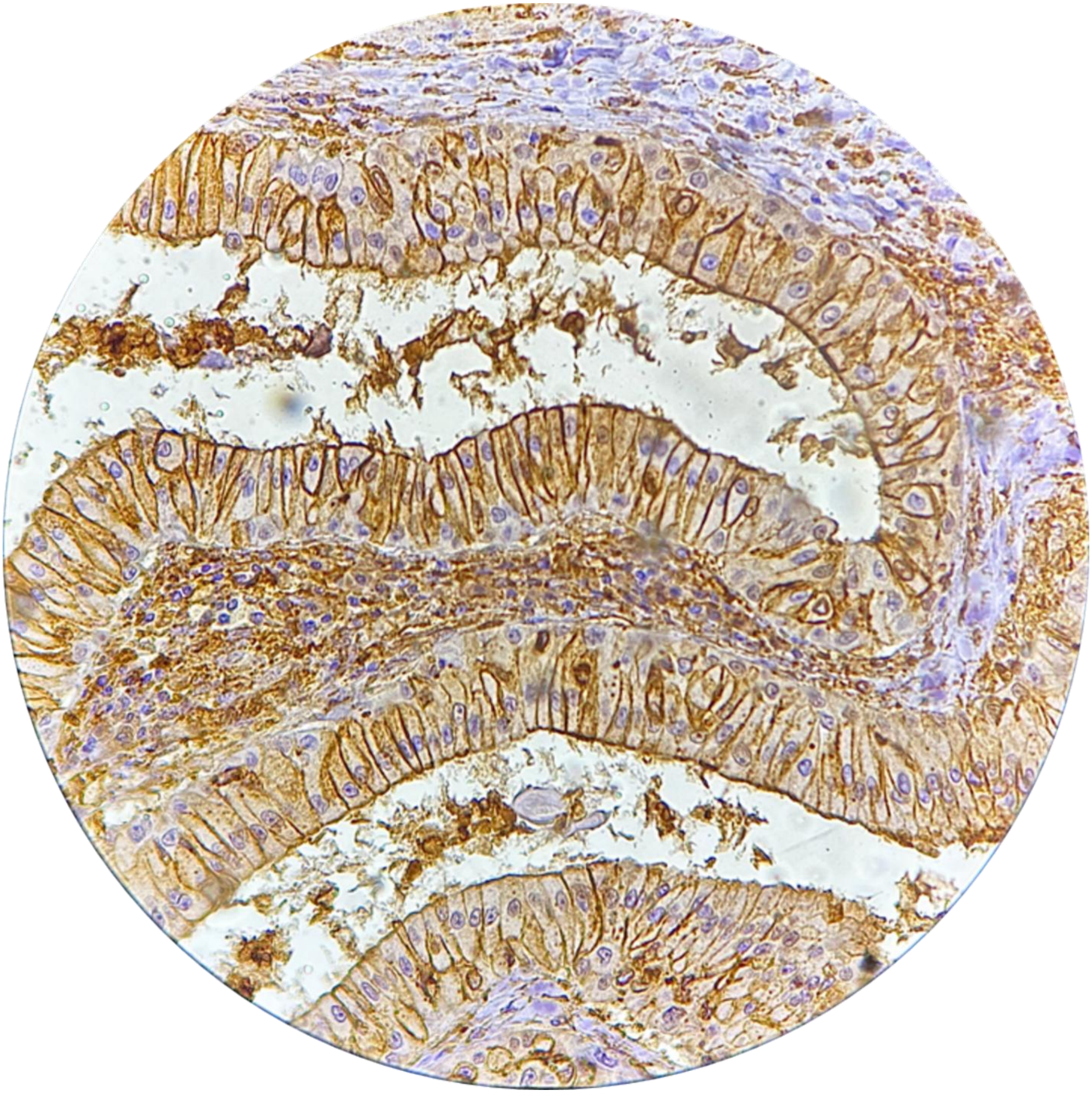

Figure S20. Complete microscopic field of the same WT section shown in Figure 1. L (IHC, x400).

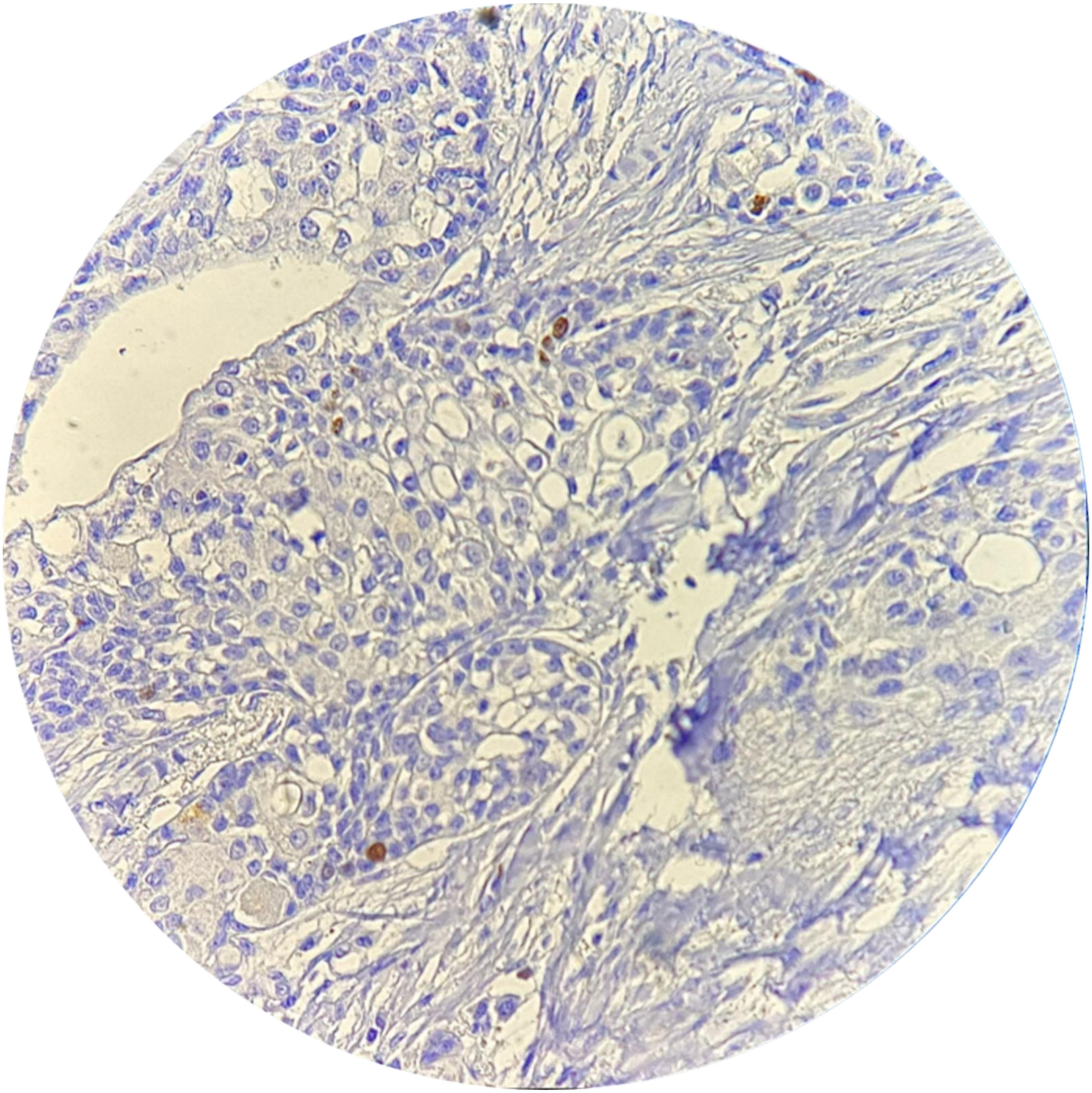

Figure S21. Complete microscopic field of the same MEC section shown in Figure 2. A (IHC, x400).

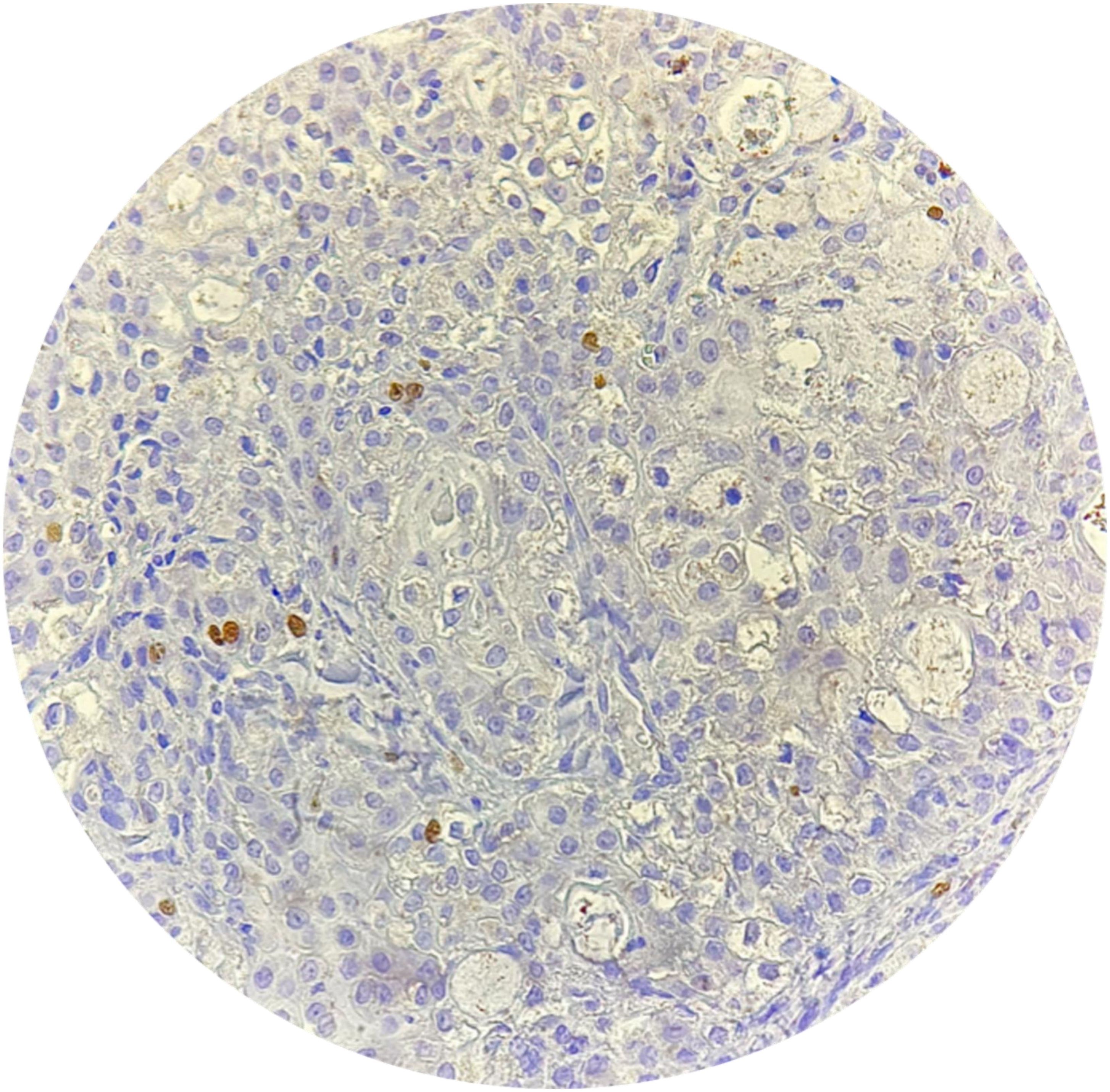

Figure S22. Complete microscopic field of the same MEC section shown in Figure 2. B (IHC, x400).

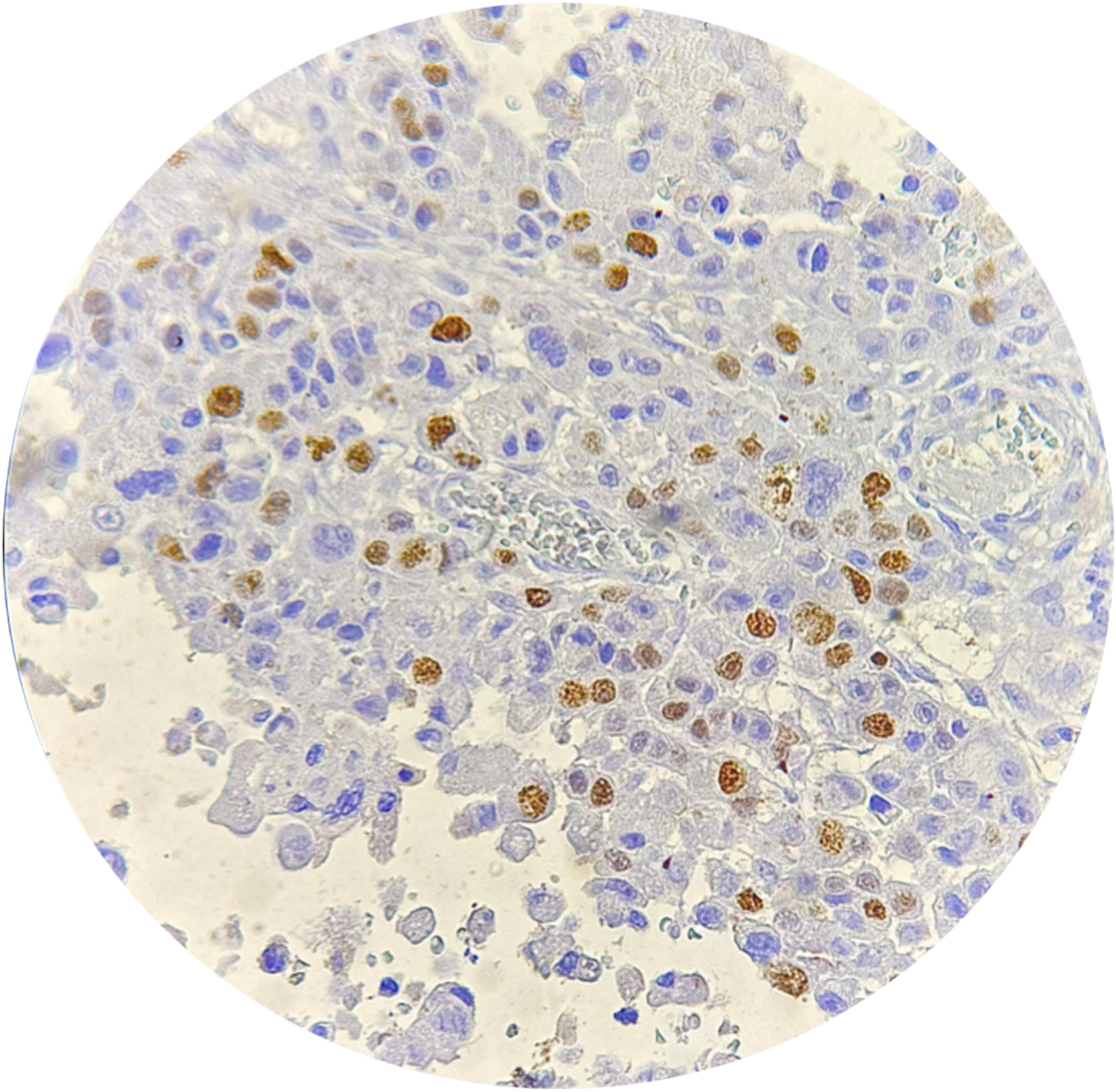

Figure S23. Complete microscopic field of the same MEC section shown in Figure 2. C (IHC, x400).

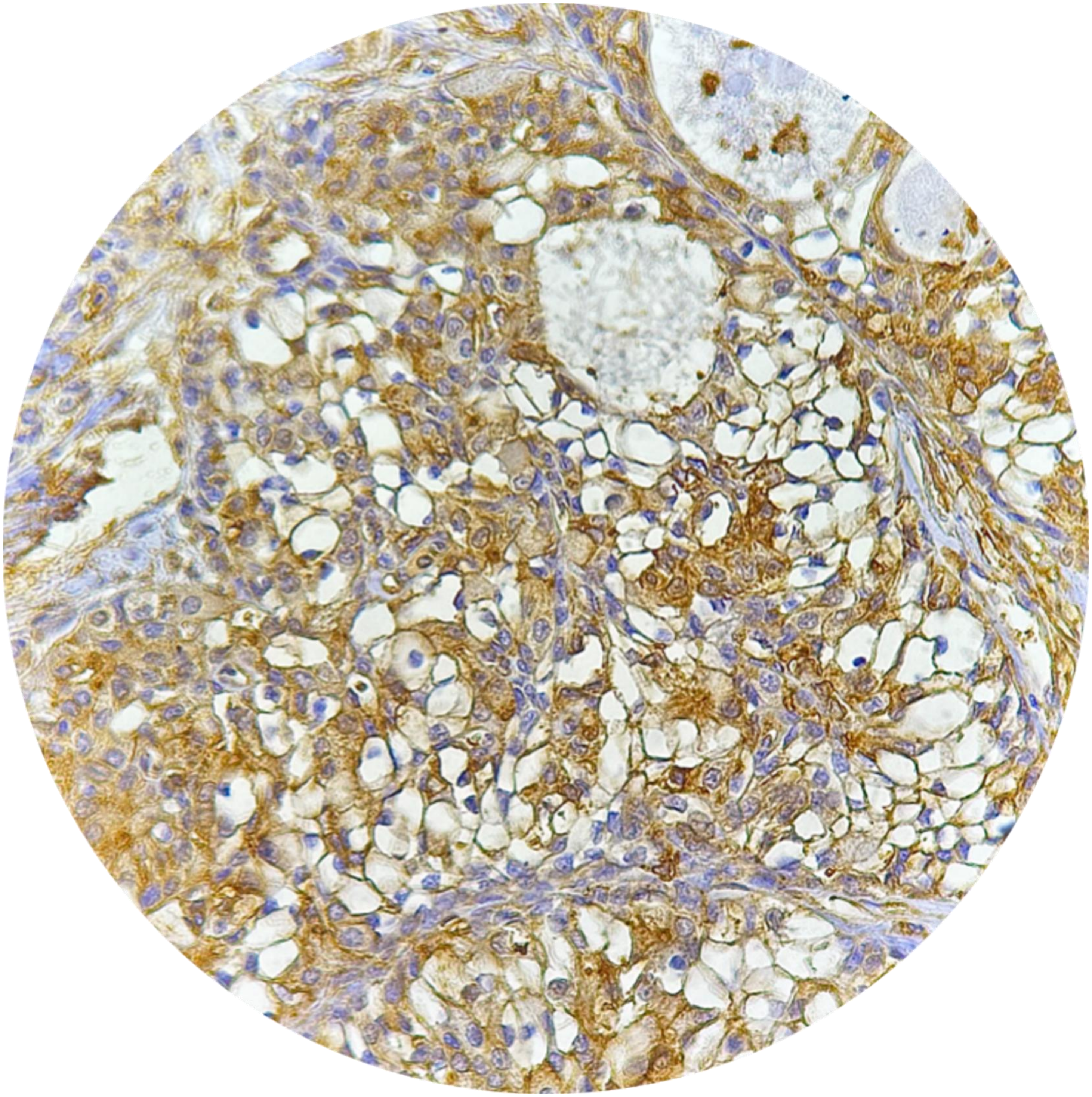

Figure S24. Complete microscopic field of the same MEC section shown in Figure 2. D (IHC, x400).

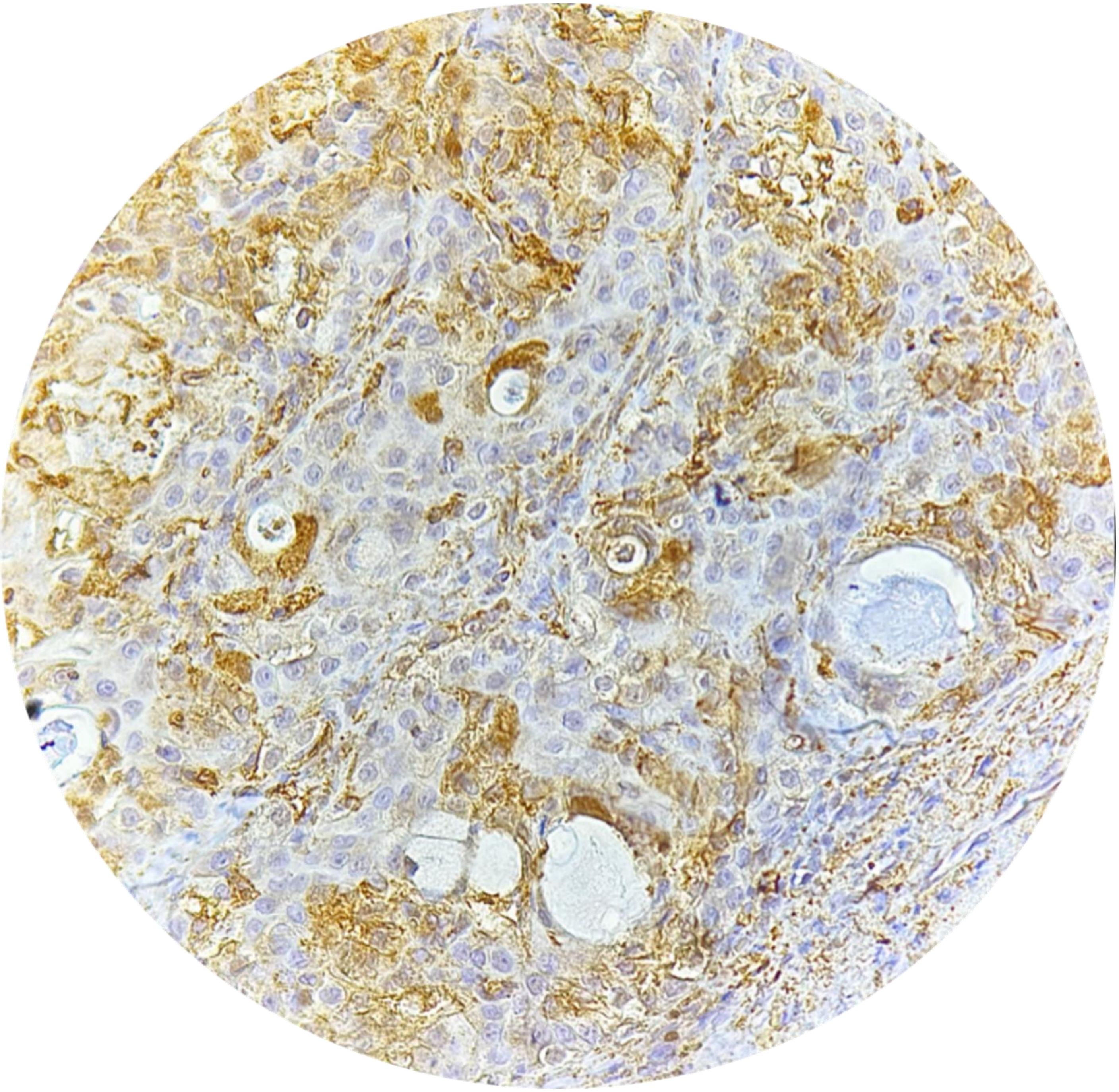

Figure S25. Complete microscopic field of the same MEC section shown in Figure 2. E (IHC, x400).

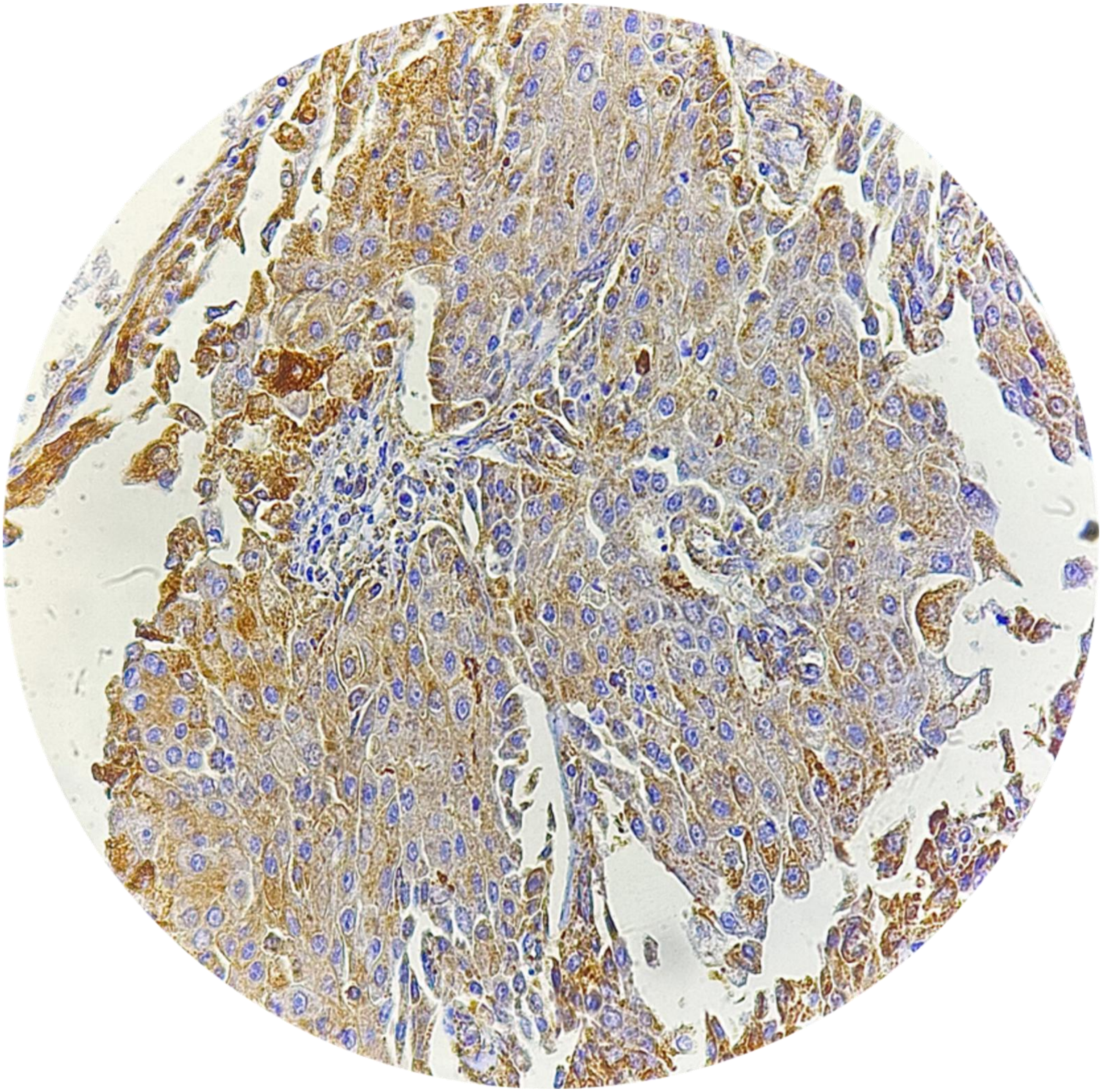

Figure S26. Complete microscopic field of the same MEC section shown in Figure 2. F (IHC, x400).

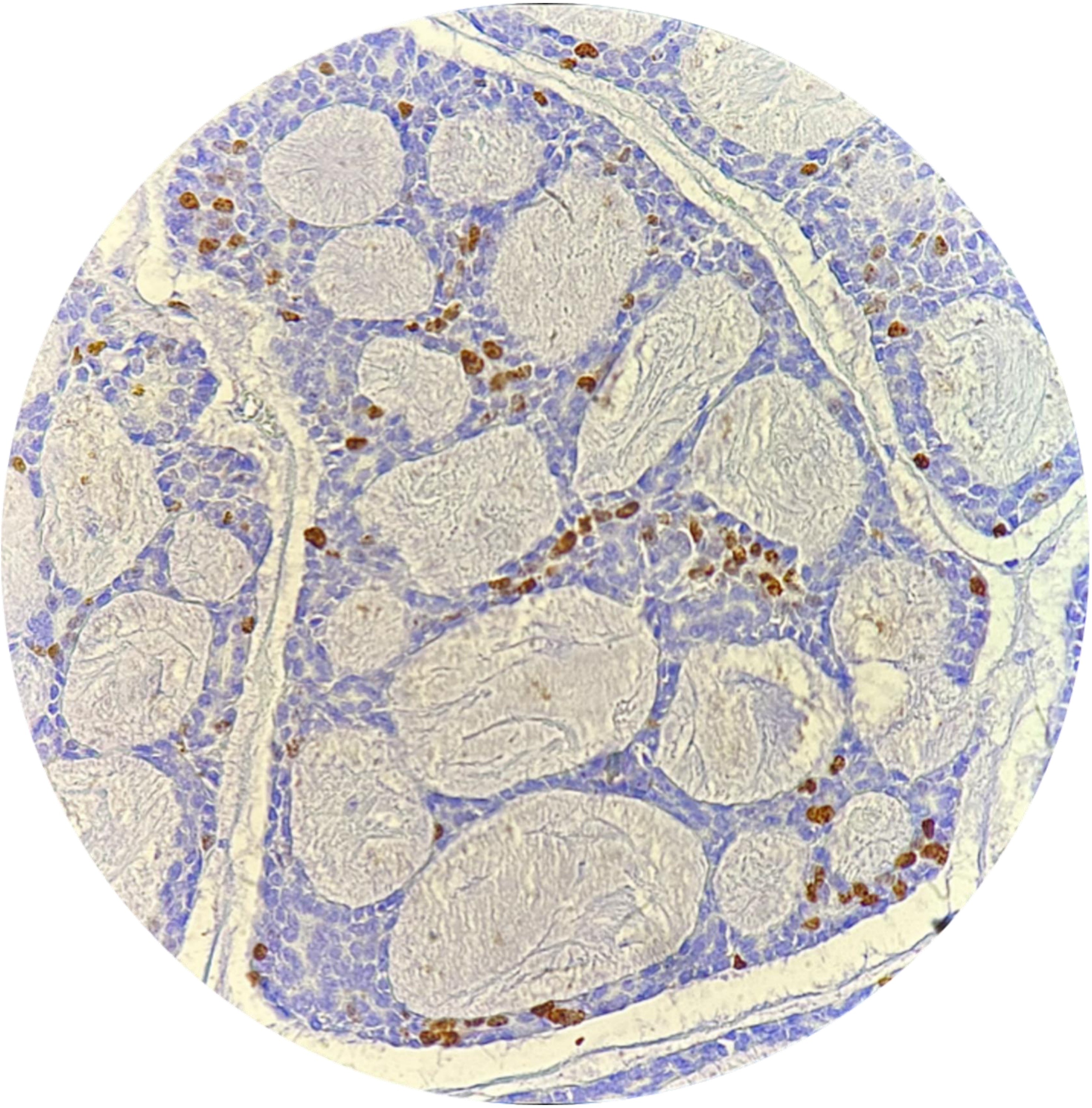

Figure S27. Complete microscopic field of the same AdCC section shown in Figure 2. G (IHC, x400).

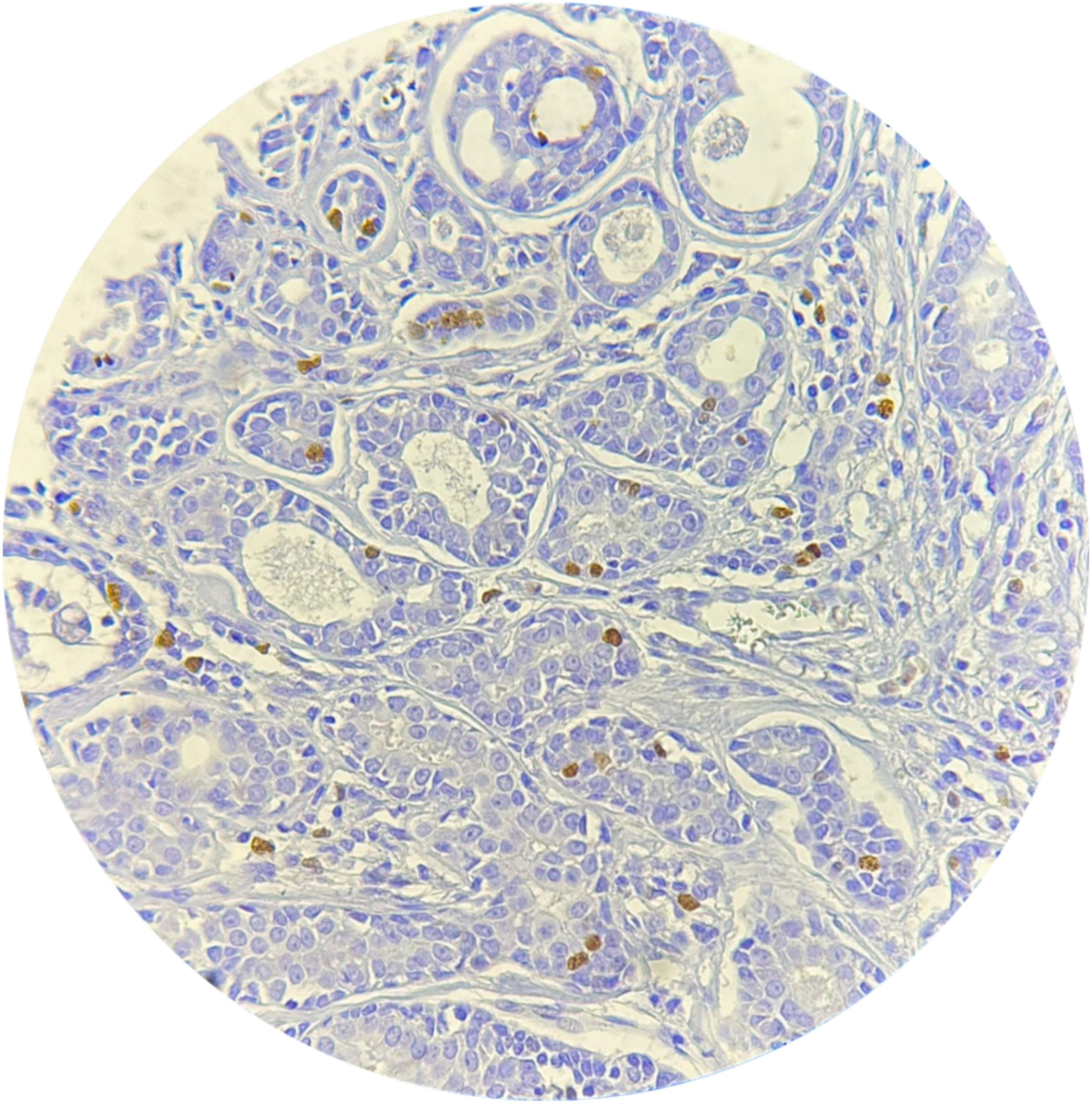

Figure S28. Complete microscopic field of the same AdCC section shown in Figure 2. H (IHC, x400).

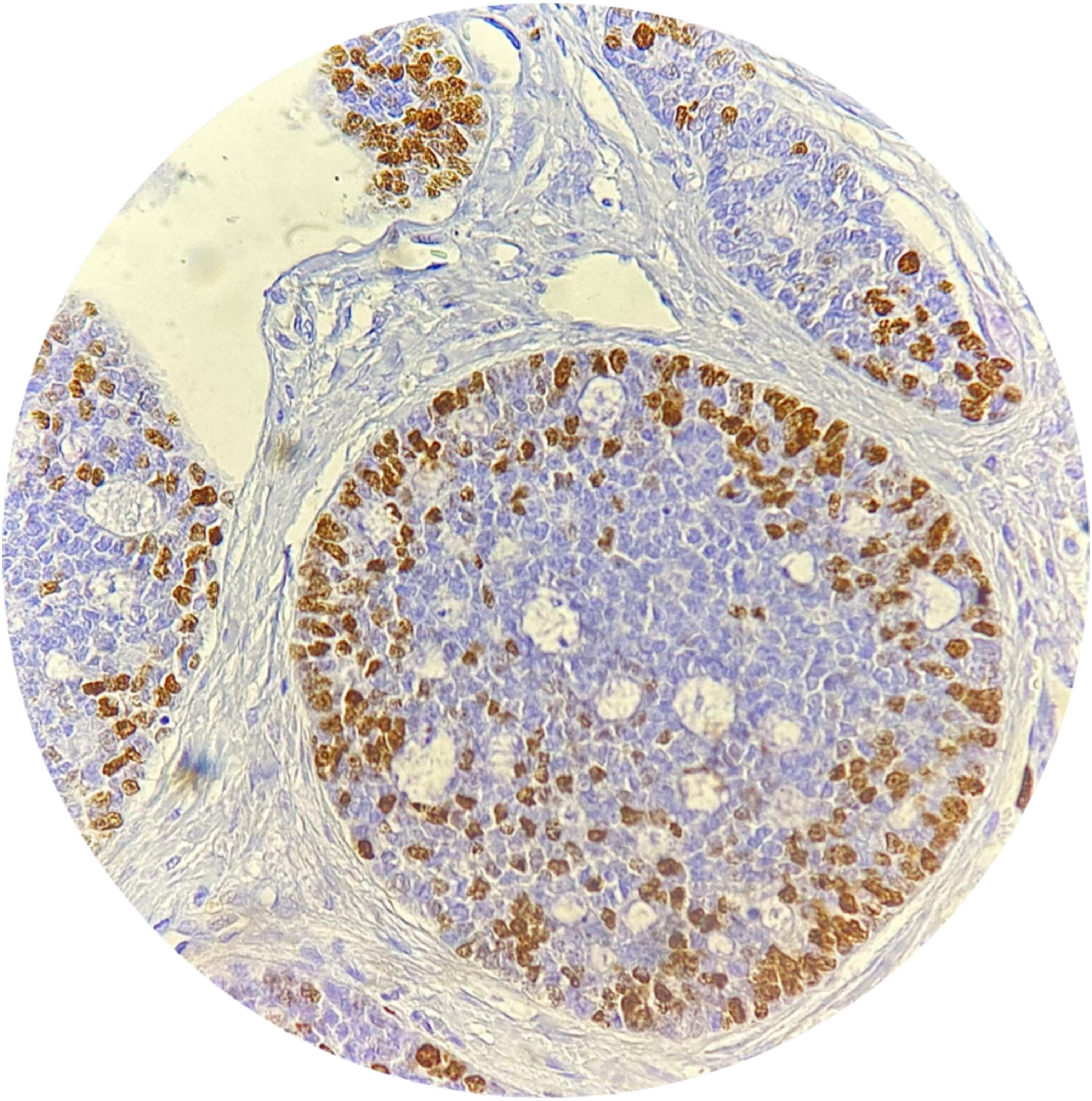

Figure S29. Complete microscopic field of the same AdCC section shown in Figure 2. I (IHC, x400).

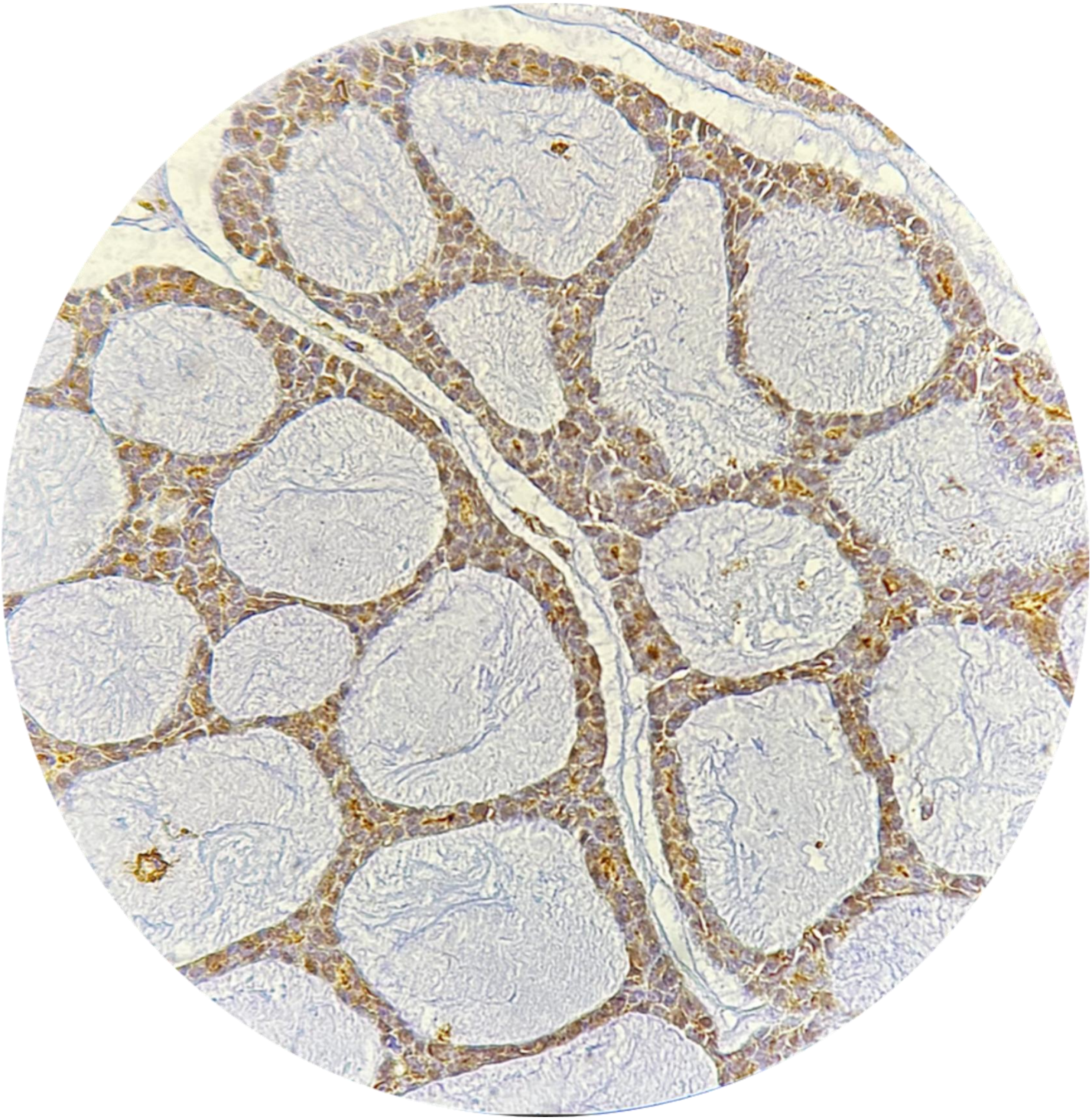

Figure S30. Complete microscopic field of the same AdCC section shown in Figure 2. J (IHC, x400).

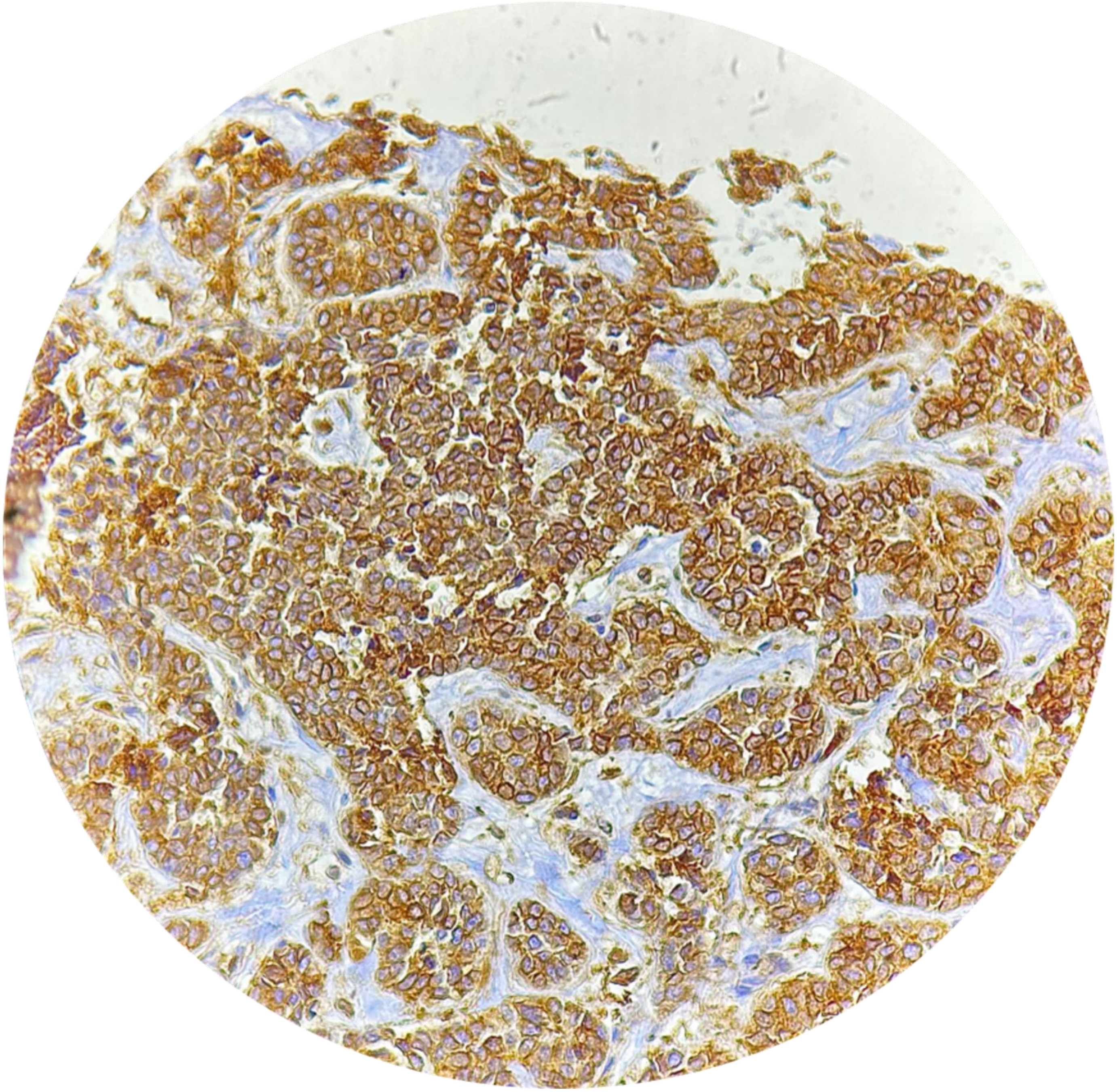

Figure S31. Complete microscopic field of the same AdCC section shown in Figure 2. K (IHC, x400).

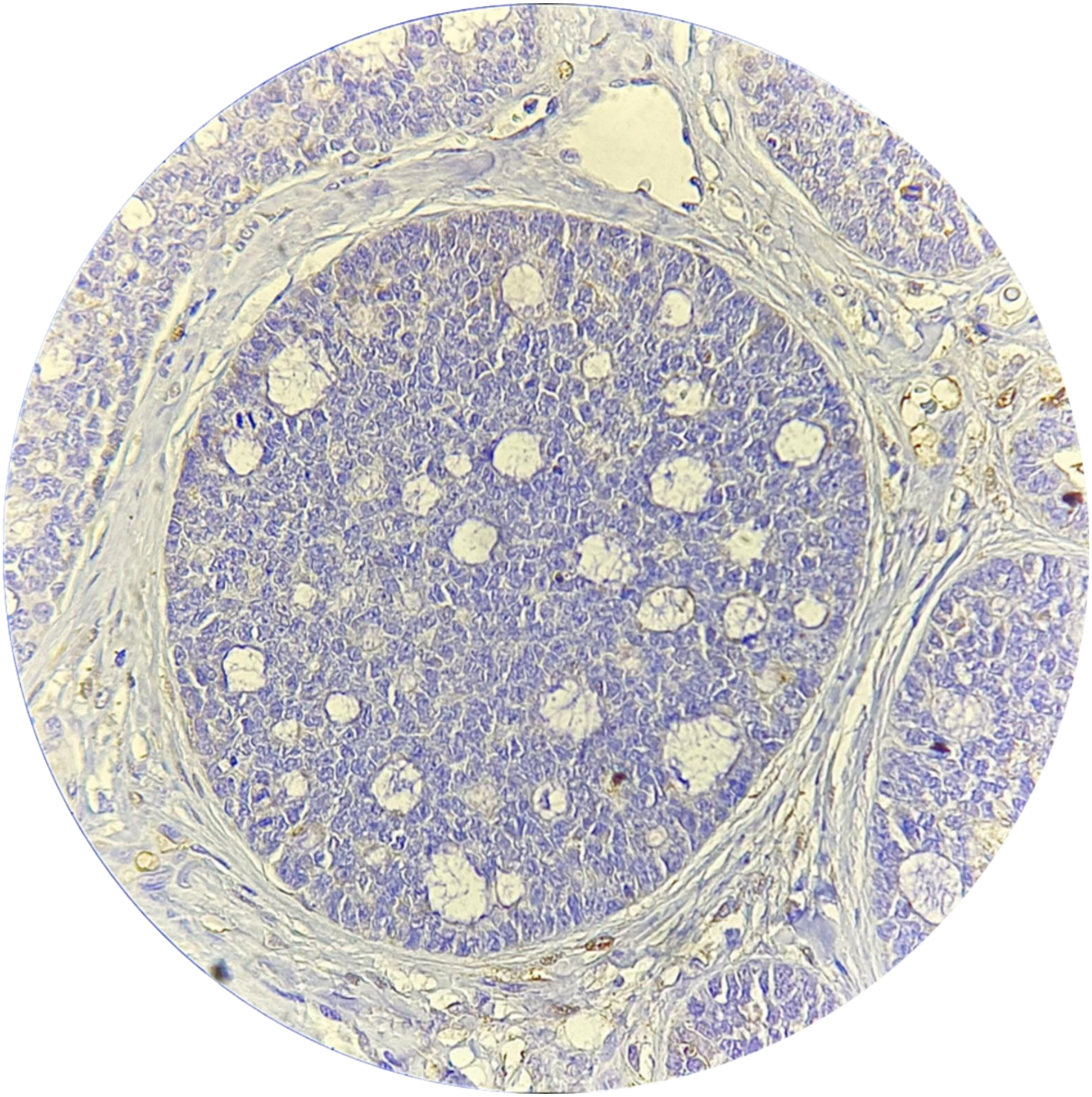

Figure S32. Complete microscopic field of the same AdCC section shown in Figure 2. L (IHC, x400).
